# The Prospects of gene introgression from crop wild relatives into cultivated lentil for climate change mitigation

**DOI:** 10.3389/fpls.2023.1127239

**Published:** 2023-03-10

**Authors:** Vijay Rani Rajpal, Apekshita Singh, Renu Kathpalia, Rakesh Kr. Thakur, Mohd. Kamran Khan, Anamika Pandey, Mehmet Hamurcu, Soom Nath Raina

**Affiliations:** ^1^ Department of Botany, Hansraj College, University of Delhi, Delhi, India; ^2^ Amity Institute of Biotechnology, Amity University Uttar Pradesh, Sector 125, Noida, U.P., India; ^3^ Department of Botany, Kirori Mal College, University of Delhi, Delhi, India; ^4^ Department of Soil Science and Plant Nutrition, Faculty of Agriculture, Selcuk University, Konya, Türkiye

**Keywords:** crop wild relatives (CWRs), lentils, climate change, crop improvement, biotic and abiotic stresses, omics-approaches, gene introgression, molecular breeding

## Abstract

Crop wild relatives (CWRs), landraces and exotic germplasm are important sources of genetic variability, alien alleles, and useful crop traits that can help mitigate a plethora of abiotic and biotic stresses and crop yield reduction arising due to global climatic changes. In the pulse crop genus *Lens*, the cultivated varieties have a narrow genetic base due to recurrent selections, genetic bottleneck and linkage drag. The collection and characterization of wild *Lens* germplasm resources have offered new avenues for the genetic improvement and development of stress-tolerant, climate-resilient lentil varieties with sustainable yield gains to meet future food and nutritional requirements. Most of the lentil breeding traits such as high-yield, adaptation to abiotic stresses and resistance to diseases are quantitative and require the identification of quantitative trait loci (QTLs) for marker assisted selection and breeding. Advances in genetic diversity studies, genome mapping and advanced high-throughput sequencing technologies have helped identify many stress-responsive adaptive genes, quantitative trait loci (QTLs) and other useful crop traits in the CWRs. The recent integration of genomics technologies with plant breeding has resulted in the generation of dense genomic linkage maps, massive global genotyping, large transcriptomic datasets, single nucleotide polymorphisms (SNPs), expressed sequence tags (ESTs) that have advanced lentil genomic research substantially and allowed for the identification of QTLs for marker-assisted selection (MAS) and breeding. Assembly of lentil and its wild species genomes (~4Gbp) opens up newer possibilities for understanding genomic architecture and evolution of this important legume crop. This review highlights the recent strides in the characterization of wild genetic resources for useful alleles, development of high-density genetic maps, high-resolution QTL mapping, genome-wide studies, MAS, genomic selections, new databases and genome assemblies in traditionally bred genus *Lens* for future crop improvement amidst the impending global climate change.

## 1 Introduction

Climate change is a global threat to food and nutritional security (Leisner, 2020; Shahzad et al., 2021) as predicted by the intergovernmental panel on climate change (IPCC) (Climate.gov, 2022). The expected average global temperature rise between 2°C and 3°C by 2100 is anticipated to severely impact both abiotic and biotic components of the environment (Tito et al., 2018; Juroszek et al., 2020; Skendžić et al., 2021; Pielke et al., 2022), resulting in impacts on soil nutrients and other ecological resources, as well as the growth, abundance, distribution, physiology and phenology of a wide range of species (Shao and Halpin, 1995; Tollefson, 2020). Agriculture is particularly vulnerable to the effects of climate change, with significant yield losses due to heat and drought waves and the emergence of new diseases. The inconsistent precipitation, water deficit, extreme temperatures and sodicity have been among the most devastating stresses that have caused enormous reduction in crop productivity (Rajpal et al., 2019a; Rajpal et al., 2019b; Zeroual et al., 2023). Many modelling studies conducted in multiple countries and agro-climatic zones have predicted large-scale reduction in agricultural productivity, habitat loss, distribution, range shifts and even extinction of species coupled with climate change (Bellard et al., 2012; Iizumi et al., 2018; Gupta and Mishra, 2019; Román-Palacios and Wiens, 2020; Zilli et al., 2020; Kadiyala et al., 2021; Lychuk et al., 2021; Affoh et al., 2022; Ait-El-Mokhtar et al., 2022; Gordeev et al., 2022; Nguyen and Scrimgeour, 2022; Ntiamoah et al., 2022 **)** and the risks being exacerbated in species with narrow distribution range and/or genetic base (Dubos et al., 2022; Galushko and Gamtessa, 2022). Besides mitigating commercial cultivars to adapt to the changing climates, there is a pressing need to enhance crop productivity to feed the world’s ever-growing population which is expected to reach 9 billion by the year 2050. This can be achieved by increasing the rate of genetic gains using novel technologies enabling the crop breeding reduction, increasing genetic gains accuracy and using wide genetic diversity. Breeding climate-smart crop varieties that can withstand multiple stresses in field conditions, therefore, is the focus of modern plant breeding research worldwide. The identification and availability of stress-responsive genes and loci, which is a prerequisite for implementing these strategies has also become a thrust area of research.

In this context, the crop wild relatives (CWRs), landraces and exotic germplasm serve as important reservoirs of useful genes for resistance to insect pests, diseases and various abiotic stresses. A plethora of published reports has clearly demonstrated that a variety of traits like increased resistance against late blight, grassy stunt disease, drought and heat tolerance, increased nutritional value and productivity (Brar and Khush, 1997; Bamberg and Hanneman, 2003; Sheehy et al., 2005; Song et al., 2014; Janzen et al., 2019; Wang et al., 2019; Hao et al., 2020; Gramazio et al., 2021; Quezada-Martinez et al., 2021 **)** in diverse crops including wheat, potato, soybean, mustard and rice have been achieved by introgressing useful genes from the CWRs gene pools into the commercial cultivars. Introgression breeding has given rise to improved cultivars in many leguminous species also such as peanut, urd bean, common bean mung bean, chick pea, pigeon pea and lentils (Singh et al., 1997; Singh et al., 2013; Tullu et al., 2013; Kahraman et al., 2015; Ogutcen et al., 2018; Kumar et al., 2021; Khan et al., 2022). The importance of CWRs in the breeding of novel cultivars with improved acclimatization ability to various biotic and abiotic stresses, and in broadening the genetic base of modern crops has been very well established. Therefore, efforts have been done globally to characterize and conserve these important genetic treasures for future crop protection and sustenance of agri-food systems (Jarvis et al., 2008; Rajpal et al., 2016a; Coyne et al., 2020; Dissanayake et al., 2020; García-García et al., 2021; Quezada-Martinez et al., 2021; Pratap et al., 2021; Renzi et al., 2022; Rajandran et al., 2022).

The genus *Lens* (2n=2x=14), an important source of food, fodder and dietary protein is one of the most important members of the family Fabaceae (Schaefer et al., 2012). The genus has undergone many taxonomical revisions and according to the most accepted classification system, it consists of seven taxa, *viz. L. culinaris* ssp. *culinaris*; *L. culinaris* ssp. *orientalis*; *L. culinaris* ssp. *odemensis; L. ervoides; L. culinaris* ssp. *tomentosus*; *L. lamottei* and *L. nigricans* (Ferguson et al., 2000; Ferguson and Erskine, 2001). *L. culinaris* ssp. *culinaris* commonly known as lentil is the only cultivated species of the genus with *L. culinaris* ssp. *orientalis* and *L. nigricans* being its most closely related and distant progenitors, respectively (Wong et al., 2015).

Lentil (*L. culinaris* ssp. *culinaris*), an annual, herbaceous and self-pollinated old world crop is believed to have been domesticated around 8500 BC in Syria and Turkey (Hansen and Renfrew, 1978; Cubero, 1981; Harlan, 1992; Bahl et al., 1993; Zohary and Hopf, 2000). It originated in the Near East and Asia Minor (Ladizinsky, 1979; Zohary and Hopf, 1988; Ferguson et al., 2000) and has since spread to other regions such as North Africa, South Asia, Central and Southern Europe, North America, and Oceania after its origin from Eastern Fertile Crescent (Duke, 1981; Ahmad et al., 1997). Lentil is now widely cultivated in a range of climates and elevations and is the 3^rd^ most important grain legume after chickpea and pea. It is a dual-purpose crop with its grains being a source of high dietary protein and straw being a valuable livestock feed. There has been a significant increase in global yield potential for lentil over the past 25 years (FAOSTAT, 2019) leading to an increase in global production from 0.85 to 6.53 metric tonnes (FAOSTAT, 2020). Canada is world’s largest lentil producer (48% of world’s production) and exporter (64%. of global lentil exports), while India is the second largest producer (with 15.7% of world’s production) but the largest importer of lentil due to high consumption and low productivity (Dissanayake et al., 2020; Rajendran et al., 2021; http://www.fao.org/faostat/en/#data/QC; http://www.fao.org/faostat/en/#data/TP, Guerra-García et al., 2021).

The successful breeding and genetic enhancement of crops depend on the availability of genetic diversity in their gene pools, identification and characterization of the novel alleles and detailed crossability data for selecting relevant taxa as parents (Rajpal et al., 2016a; Rajpal et al., 2016b). On the basis of crossability data, the species of genus *Lens* have been grouped into three gene pools, with the primary gene pool being represented by *Lens culinaris* ssp*. culinaris, L. culinaris* ssp*. orientalis*, and *L. odemensis.* The secondary, and tertiary gene pools are represented by two species each *L. ervoides*, *L. nigricans* and *L. lamottei* and *L. tomentosus*, respectively (Ladizinsky, 1999; Muehlbauer and McPhee, 2005; Fratini and Ruiz, 2006). These gene pools are the reservoirs of useful crop traits such as resistance to various pathogens and other phenological and agronomic traits (Gupta and Sharma, 2006; Cristobal et al., 2014) that can be transferred to cultivated lentils.

Traditionally, lentil breeding has been undertaken through extensive germplasm screening which has allowed selection and release of superior cultivars such as varieties BARI M4-M8 (Bangladesh) (Kumar et al., 2021) and ILL 404 (Nepal) (Materne and McNeil, 2007) with improved yield and disease resistance for commercial cultivation. An exotic variety ‘Percoz’ has resulted in many improved Indian cultivars Angoori, Narendra M1, and VL Masoor 507 (Kumar et al., 2013). However, intensive breeding and domestication have led to a narrow genetic base and reduced yield of local lentil cultivars, which limits the prospects of further increasing crop productivity through selections. Based on morphological differences, the cultivated lentil species *L. culinaris* encompass the small-seeded (*microsperma*) and large-seeded (*macrosperma*) groups (Singh et al., 2020). In India, traditionally grown lentil belongs to ‘microsperma’ (pilosae type), which has a narrow genetic base, low seedling vigor, pod set and harvest index and increased rate of flower drop. It is also poor in dry matter accumulation and lacks resistance to abiotic and biotic stresses (Ferguson et al., 1998; Kumar et al., 2004; Khazaei et al., 2016; Zeroual et al., 2023). To achieve enhanced genetic gains in lentil breeding, the identification of new target traits from CWRs and their introgression into cultivated taxa is desired in order to broaden the genetic base of cultivars. This can be accomplished by deploying additional alleles from alien and secondary and tertiary gene pools. Recent advances in large-scale genome analyses, such as next generation sequencing (NGS), high throughput genotyping (HTG) and high throughput phenotyping (HTP) have added to the breadth of genetic diversity, development of genomic resources databases and knowledge on phylogenetics in the genus *Lens.* This information can be used for precise and efficient molecular genetic improvement and enhancement programs of lentils (Kumar et al., 2021; Pratap et al., 2021; Hussain et al., 2022; Salaria et al., 2022; Salgotra and Stewart, 2022; Singh et al., 2022a; Tiwari et al., 2022; Civantos-Go´ mez et al., 2022; Roy et al., 2023; Zeroual et al., 2023 **)** similar to what has been achieved in major crops such as rice, wheat and maize (Yoshino et al., 2019; Mishra et al., 2021).

The present Review has compiled information on the available genetic and genomic resources, genotyping efforts, genetic maps and databases, marker-assisted and genomic selections, identification of QTLs, ESTs, genes associated with desired crop traits and genome assemblies in lentil and its CWRs. This collation will aid in understanding the spectrum of diversity available for introgression and the development of elite lentil germplasm with desired productivity levels for future food and nutritional security and adaptability to changing climates.

## 2 Gene Pools, phylogenetic relationships, and domestication of lentil

Lentil is a self-pollinated, diploid (2n=2x = 14) species with a C DNA value of 4.2 pg (Arumuganathan and Earle, 1991; Singh et al., 2018). The taxonomy of genus *Lens* at the species and subspecies levels has been quite contentious (Van Oss et al., 1997; Ferguson et al., 2000; Fratini and Ruiz, 2006; Suvorova, 2014; Koul et al., 2017). The most recent classification system (Wong et al., 2015; Koul et al., 2017) recognizes seven taxa in the genus grouped into four genepools: *L. culinaris, L. orientalis* and *L. tomentosus* in the primary genepool; *L. odemensis*, *L. lamottei* in the secondary genepool; and one species each *L. ervoides* and *L. nigricans* in the tertiary and the quaternary gene pools, respectively. Despite these reorganizations at taxonomic level, it is generally agreed that *L. culinaris* ssp. *orientalis* is the most closely related wild progenitor of *L. culinaris* ssp. *culinaris*, while the most distantly related species *L. nigricans* has a distinct gene pool (Reddy et al., 2009; Wong et al., 2015; Liber et al., 2021 **).** Although viable hybrid formation has been reported between *L. culinaris* ssp. *orientalis* and *L*. *odemensis* (Ladizinsky et al., 1984; Abbo and Ladizinsky, 1994; Fratini et al., 2004; Fratini and Ruiz, 2006; Muehlbauer et al., 2006), the fertility of the hybrids may be affected by chromosomal rearrangements (Ladizinsky et al., 1984; Ladizinsky, 1979 **).** Crosses are also possible between the cultivated lentil, *L. culinaris* and the species belonging to the other gene pools, but hybrids may be sterile owing to chromosomal rearrangements that aborts the hybrid embryos at a high rate (Abbo and Ladizinsky, 1991; Ladizinsky, 1993; Abbo and Ladizinsky, 1994; Gupta and Sharma, 2005). *In vitro* embryo rescue methods are used to overcome these barriers (Fratini and Ruiz, 2006; Fratini and Ruiz, 2011; Kumar et al., 2014).

Studies have reported a close relationship between *L. odemensis*, *L. nigricans* and *L*. *culinaris* ssp. *orientalis* based on morphological markers (Fratini et al., 2006), however, other studies using morphological features and molecular markers suggest the need for revisions in the taxonomic status of *L. culinaris* ssp. *odemensis* and *L. tomentosus* which have distinct morphological features and karyotypes (Ladizinsky, 1997; Van Oss et al., 1997; Koul et al., 2017 **)**. These differences in the karyotypes might contribute to the reproductive isolation between *Lens* species, even though they share the same diploid chromosome number (Muehlbauer and McPhee, 2005).

Further, three major cultivated lentil groups have been identified by Khazaei et al. (2016) based on studies on lentil accessions from 54 countries reflecting the world’s Mediterranean, northern temperate and south Asian (sub-tropical savannah) agro-ecological zones. Four major clusters have also been revealed by Dissanayake et al. (2020) with the taxa grouped as *L. culinaris*/*L. orientalis in* cluster 1; cluster 2 with *L. odemensis*/*L. lamottei*; and two species *L. ervoides* and *L. nigricans* clustered separately. Studies by Pavan et al. (2019) showed correlation between assessment of seed size and early flowering traits, genetic clustering and geography in Mediterranean germplasm. Cultivated and wild lentil accessions showed little correlation in their geographical origins. These reports indicate that present-day lentil diversity has been articulated by both natural and artificial selection (Liber et al., 2021).

## 3 World lentil genetic resources

Worldwide, gene banks hold a large number of 58,405 *Lens* accessions spread across 103 countries. The International Centre for Agricultural Research in the Dry Areas (ICARDA) maintains the largest collection of 14,577 accessions, including 11,405 landraces, 2,580 breeding lines and 612 wild accessions from 26 countries (Kumar et al., 2015; Guerra-García et al., 2021). Other large germplasm collections of lentil are maintained by the Australian Grains Gene (AGG) bank (6,218 accessions), the European Cooperative Programme for Plant Genetic Resources (4,598 accessions), the USDA Agricultural Research Service, USA (3,247 accessions), the Seed and Plant Improvement Institute of Iran (3,000 accessions), the Vavilov Institute, Russia (2,598 accessions), and Plant Gene Resources of Canada (1,150 accessions). In India, the ICAR-National Bureau of Plant Genetic Resources (NBPGR) of India maintains 2537 accessions, while, Indian Institute of Pulses Research (IIPR), Kanpur, maintains 71 accessions from wild species and 117 landraces of the cultigen from the Mediterranean region (Kumar et al., 2015; Singh and Chung, 2016; Malhotra et al., 2019). The distribution of lentil world collections is listed in **Table 1**.

**Table 1:** List of World Germplasm Collections in Lentil.

Genebank/Institute	Accessions		
	Total number	Wild taxa	Land races/cultivars
International Centre for Agricultural Research in Dry Areas (ICARDA), Syria	14577	612	11405
Australian Temperate Field Crops Collection, Australian Grains Gene bank (AGG)	6218	250	3037
United States of Department of Agriculture,USA	3247	52	454
Seed and Plant Improvement Institute, Iran	3000	270	360
Vavilov Institute, Russia	2598	285	1740
National Bureau of Plant Genetic Resources, India	2537	108	1871
Plant Gene Resources of Canada	1150	195	644
Plant Genetic Resource Department Aegean Agricultural Research Institute, Turkey	1095	10	1084
General Commission for Scientific Agricultural Research, Syria	1072	75	407
Research Centre for Agro-Botany, Hungary	1061	42	31

Keeping in view the global mandate for lentil improvement, accessions of different wild species of lentil are screened at various research institutions such as ICARDA, and IIPR for various biotic and abiotic stresses, as well as agro-morphological traits. Further, hybridization programs involve crossing ‘microsperma’ and ‘macrosperma’ lentils (Erskine et al., 1998) to produce promising germplasm for lentil breeding programs in South Asia (Sarker and Erskine, 2006; Sarker et al., 2010). The introduction of exotic germplasm of macrosperma variety ‘Precoz’ with early flowering trait has led to the development of improved cultivars with large seeds, short duration and rust resistance (Singh et al., 2006; Asghar et al., 2010). There are many cultivars that have been developed and released in India using promising breeding lines developed at ICARDA (Dixit et al., 2009).

A recent initiative, INCREASE (Intelligent Collections of Food Legumes Genetic Resources for European Agrofood Systems) launched in 2020 by the European Union’s Horizon (https://www.pulsesincrease.eu) aims to enhance the phenotypic and genotypic characterization of four food legumes genetic resources including lentil (García-García et al., 2019; Cortinovis et al., 2021; Guerra-García et al., 2021; Kroc et al., 2021).

To manage a large number of accessions, concept of developing ‘core’ and ‘mini core’ collections has been used to represent maximum variability in limited number of accessions (Brown, 1989 **).** While a core collection represents 10-20% (Yonezawa et al., 1995) of the total base collection of accessions in a species, a mini core collection includes 1-2% of entire collection (Zhang et al., 2012). Core collections are attractive as they represent a sizeable genetic diversity in a manageable number of accessions and have been developed in many crop species like rice, wheat, maize, and many pulses (Upadhyaya et al., 2006; Mourad et al., 2020; Vilayheuang et al., 2020; Raturi et al., 2022). In the genus *Lens*, Singh et al. (2014) analysed 405 accessions of all seven taxa with morphological and biotic resistance markers to construct a core set of 96 lentil accessions using the statistical program ‘PowerCore’. The core set was then screened for resistance to rust (*Uromyces fabae* (Grev.) Fuckel) and Powdery mildew (*Erysiphe polygoni* DC.) for three seasons under two agro-climatic conditions in India (Singh et al., 2014). Another core set of lentil accessions comprising of 170 accessions (137 Indian and 33 exotic) has been constructed based on the agro-morphological data and geographical distribution (Tripathi et al., 2021). Recently, Heineck et al. (2022) screened a part of the lentil core collection derived from single seed for resistance against *Fusarium oxysporum.* They found differences in disease severity and biomass traits among lentil accessions. Further, they used genome-wide association study (GWAS) and SNP markers to identify 11 QTLs, two pairs of which were located near putatively orthologous sequences linked to disease resistance.

## 4 Crop wild relatives (CWRs) as a source of novel variation for economically important traits

Conventional breeding has resulted in considerable genetic improvement of lentils, but productivity has become stagnant in the recent years. Utilization of divergent germplasm from crop wild relatives, landraces and exotic germplasm can broaden the genetic base with useful genetic variation and infuse the lost variability which can result in improved productivity and introgression of desirable characters in lentil (Doyle, 1988; Tanksley and McCouch, 1997; Gupta and Singh, 2009; Pratap and Gupta, 2009). Domesticated lentil has revealed very poor genetic variability compared to its related wild species *L. culinaris* ssp. *orientalis* in multiple studies (Muench et al., 1991; Mayer and Soltis, 1994; Alvarez et al., 1997; Ford et al., 1997; Alo et al., 2011). Many studies have indicated that wild *Lens* taxa show resistance to various biotic and abiotic stress conditions (Bayaa et al., 1994; Bayaa et al., 1995; Hamdi et al., 1996; Hamdi and Erskine, 1996; Gupta and Sharma, 2006). These species are a source of useful alleles for traits like resistance to key diseases, parasitic weeds and insect pests. The different sources of important crop traits in lentils are listed in **Table 2**.

**Table 2:** Wild germplasm resources for economically important traits in lentil.

Gene Pools*	Species	Traits		References
Primary	*L. culinaris* ssp. *culinaris*	Biotic Resistance	Anthracnose or *Colletotrichum truncatum* resistance	Buchwaldt et al., 2004; Shaikh et al., 2013
		Abiotic Resistance	Heat tolerance	Kumar et al., 2015
Primary	*L. culinaris* ssp. *orientalis/L. orientalis*	Biotic Resistance	*Ascochyta* blight, *Stemphylium* blight, *Fusarium* wilt, Powdery mildew Rust	Bayaa et al., 1994; Gupta and Sharma, 2006; Tullu et al., 2010; Coyne et al., 2020; Singh et al., 2020; Dadu et al., 2021
		Insect Resistance	Bruchids *Orobanche*	Fernández-Aparicio et al., 2009; Laserna-Ruiz et al., 2012
		Abiotic Resistance	Cold tolerance, salinity	Hamdi et al., 1996; Singh et al., 2017a
			Agronomic seed weight	Abbo et al., 1992; Singh et al., 2014
Primary/Secondary	*L. culinaris* ssp. *odemensis/L. odemensis*	Biotic Resistance	*Ascochyta* blight, *Stemphylium* blight, *Fusarium* wilt, Rust Powdery mildew	Bayaa et al., 1994; Gupta and Sharma, 2006; Tullu et al., 2010; Singh et al., 2014; Singh et al., 2022a; Singh et al., 2022b; Coyne et al., 2020; Dadu et al., 2021
		Abiotic Resistance	Drought	Omar et al., 2019
		Insect Resistance	*Sitona* weevil	El-Bouhssini et al., 2008
Secondary/Tertiary	*L. ervoides*	Biotic Resistance	Anthracnose *Ascochyta* blight, *Stemphylium* blight, *Fusarium* wilt, Rust	Bayaa et al., 1994; Gupta and Sharma, 2006; Tullu et al., 2006a; Fiala et al., 2009; Tullu et al., 2010; Vail et al., 2012; Coyne et al., 2020; Singh et al., 2020
		Abiotic Resistance	Drought	Gorim and Vandenberg, 2017
		Insect Resistance	Sitona weevil *Orobanche*	El-Bouhssini et al., 2008; Fernández-Aparicio et al., 2009
		Agronomic traits	Seed size	Fiala et al., 2009; Tullu et al., 2011
Primary/Tertiary	*L. tomentosus*	Biotic Resistance	*Fusarium* wilt, Powdery mildew	Singh et al., 2014; Singh et al., 2020
		Abiotic Resistance	Drought	Gorim and Vandenberg, 2017; Omar et al., 2019
Secondary/Tertiary	*L. lamottei*	Biotic Resistance	Anthracnose, *Ascochyta* blight, *Stemphylium* blight,	Bayaa et al., 1994; Gupta and Sharma, 2006; Tullu et al., 2006a; Fiala et al., 2009; Tullu et al., 2010; Podder et al., 2013; Bhadauria et al., 2017; Singh et al., 2020
		Insect Resistance	Bruchids	Laserna-Ruiz et al., 2012
Secondary/Tertiary/Quaternary	*L. nigricans*	Biotic Resistance	Anthracnose, *Ascochyta* blight, *Stemphylium* blight, *Fusarium* wilt, Rust Powdery mildew	Bayaa et al., 1994; Gupta and Sharma, 2006; Tullu et al., 2006a; Fiala et al., 2009; Tullu et al., 2010; Saha et al., 2010a Saha et al., 2010b; Podder et al., 2013, Singh et al., 2014; Singh et al., 2020
		Insect Resistance	Bruchids	Laserna-Ruiz et al., 2012
		Abiotic Resistance	Cold, drought, heat	Hamdi et al., 1996; Hamdi and Erskine, 1996; Gorim and Vandenberg, 2017
		Agronomic Traits	Pods per plant	Singh et al., 2014

* Different Genepools mentioned for species according to reports of different authors.

Lentil breeding has been laid around a systematic breeding scheme where trait specific donors and recipient cultivars can be selected (Kumar et al., 2014). Many studies have shown that alien gene introgression from exotic wild species has substantially demonstrated higher variations for productivity and its associated traits in new segregating F_2_ population (Gupta and Sharma, 2007; Singh et al., 2013). Several agronomic and other potential traits like disease resistance and biofortification have been introgressed from *L. orientalis* and *L. ervoides* into pre-bred lines from various sources by ICARDA. These improved lines are being tested in different locations and exhibit more than 40% increase in yield compared to the check (Bakaria) along with higher percentage of micronutrients and 80–100 days of short-season cycle (Kumar et al., 2019). Recently, a lot of research interest has shifted to wild *Lens* relatives for identification of useful traits.

### 4.1 CWR Gene pool as a genomic reservoir for abiotic stress tolerance

Climate change has resulted in the emergence of various abiotic stresses such as drought, sodicity, extreme temperatures (heat, cold and frost) and flooding (Rajpal et al., 2019b), which have a significant impact on agricultural productivity. The changes in temperature and rainfall together have shown about 30% yield differences in major food crops in the last few years (Zhao et al., 2017). In order to adapt to these changing conditions, it is important to identify candidate genes and genetic loci that confer the adaptive responses of plants to these stresses. CWRs have been the main targets for hunting stress-responsive genes and loci. Further, for understanding the mechanism of abiotic stress adaptation which is quantitative in nature, identification of QTLs, use of genome wide association mapping (GWAM) and transcriptomic analysis are the main targets of future research focussed on stress mitigation.

Recently, the use of genomics-assisted and molecular breeding tools along with traditional breeding have been employed to characterize the hidden diversity in lentil CWRs. Studies have found that while *L. nigricans* showed maximum tolerance to drought, *L. orientalis* may also provide sources of genes for drought tolerance across African regions with low rainfall (Gupta and Sharma, 2006 **)**. In addition, screening of wild *Lens* germplasm has indicated resistance to drought in *L. odemensis, L. ervoides, L. lamottei, L tomentosus* and *L. nigricans* (Gupta and Sharma, 2006; Gorim and Vandenberg, 2017 **)**. Many accessions of *L. odemensis, L. ervoides* and *L. orientalis* responded to drought by increased deep rooting and some responded by delayed flowering. A reduction in transpiration rates was also observed as a means of drought tolerance in *L. tomentosus*. (Fang and Xiong, 2015; Gorim and Vandenberg, 2017 **).** Other reports have also highlighted the potential of lentil CWRs with significant differences in morphology of root traits for fine root distribution, variability in the number of nodules, and root biomass proportion in each soil layer (Gorim and Vandenberg, 2017). Omar et al. (2019) analysed drought tolerance in elite lentil varieties crossed with the CWRs. The drought tolerance was linked to cell membrane stability, root to shoot ratio increment, pubescent leaves, relative leaf water content, and reduced transpiration and wilting. Sanderson et al. (2019) with a focus to study disease resistance and tolerance to drought analysed recombinant inbred lines (RILs) in crosses of lentil cultivars with wild species *L. orientalis, L. ervoides* and *L. odemensis*, in the lentil pre-breeding project at ICARDA. These studies aimed to develop drought tolerance in lentils through identification of key drought traits by generating genetic markers for mapping in lentil and CWRs for breeding programs. This wide variation in responses to drought across the lentils indicates that wild species relatives will be important for future lentil development depending upon the successful crossing resulting in viable hybrids between the wild and cultivated species.

To understand the adaptation strategies to alkalinity stress tolerance in lentil, the morphological, anatomical, biochemical and transcriptomics features were compared between a tolerant and sensitive cultivar to show that the secondary metabolism and ABA signaling contributed towards alkalinity stress tolerance in lentil (Singh et al., 2022a). The lentil variety PDL-1 shows significant alkalinity tolerance and has the potential to be used in genetic improvement programs of lentil (Singh et al., 2022a). Efforts have also been done to identify the genes for cold tolerance (Hamdi et al., 1996) and salinity tolerance (Singh et al., 2017b) in *L. culinaris* ssp. *orientalis.* Rubio Teso et al. (2022) applied the predictive characterization model approach in *Lens* species based on the method of environmental filtering (Thormann et al., 2014) to identify lentil populations potentially tolerant to multiple abiotic stresses such as salinity, drought and water-logging in four wild taxa of *Lens* (*L. orientalis, L. ervoides, L. lamottei* and *L. nigricans).*

### 4.2 CWR Gene pool for biotic stress resistance

Climate change has resulted in the evolution of novel insects, nematodes, herbivores, microbial pathogens, and weeds, which limit the full potential of crop growth and reproduction, causing heavy productivity losses. Understanding the complex arrays of defense mechanisms and networks involving biotic stress resistance requires further research efforts. The elucidation of the regulating mechanisms is key to the identification of stress resistance genes. Exploration of CWRs with advanced genome dissecting tools has resulted in meaningful results in the form of identification of novel stress-responsive genes.

Most of the wild *Lens* species are reservoirs of genes conferring resistance to various pathogens and insects pests. *L. lamottei* and *L. ervoides* have shown a high level of resistance toward *Stemphylium* blight (Podder et al., 2013). Similarly, a significant level of resistance is shown by *L. odemensis* followed by *L. ervoides* accessions against *Sitona* weevil (El-Bouhssini et al., 2008). Some related wild *Lens* taxa have also shown potential for their usefulness in cultivated crop breeding programs exhibiting combined resistance to *Fusarium* wilt or anthracnose diseases (Bayaa et al., 1995; Gupta and Sharma, 2006; Tullu et al., 2006a; Tullu et al., 2010; Polanco et al., 2019; Singh et al., 2020).

To select resistant lentil population from wild taxa, a calibration method was developed and applied for the selection of populations of wild species for showing potential resistance to broomrape lentil rust and other rust diseases using a total of 204 and 351 *Lens* accessions, respectively (Rubio Teso et al., 2022).

### 4.3 CWR Gene pool for other agronomic traits

Lentil CWRs have been screened to reveal many other useful traits that can serve as important genomic resources for future breeding programs, allowing breeders to develop new culivars with improved traits. A collection of 405 related wild *Lens* species accessions were used to select promising 96 wild lentil accessions and were validated for target traits under multiple locations for establishing their use as stable donors in breeding programs (Singh et al., 2020). *L. ervoides* has been identified as a promising source of genes or alleles for traits such as growth habit, phenology, plant biomass, and seed traits (Tullu et al., 2011; Tullu et al., 2013; Kumar et al., 2014). A wide range of variation was observed for these different traits in related wild species of *Lens* globally representing various countries (Kumar et al., 2014). Quality traits like micronutrients (Sen Gupta et al., 2016; Kumar et al., 2018) raffinose and prebiotics among others (Tahir et al., 2011) also showed significant diversity in wild *Lens* species. Furthermore, interspecific populations generated from wide crosses between ‘*L. culinaris* ssp*. culinaris* x *L. ervoides’* resulted in major increase in traits for yield contribution (Tullu et al., 2011). Accessions with sources of genes for early growth have been identified in order to induce earliness into lentil cultivars with required genetic background. These include accessions of *L. culinaris* ssp*. culinaris* and accession ‘ILWL 118’ of *L. culinaris* ssp*. orientalis* that can potentially donate to the genetic enhancement program of lentil (Tyagi and Sharma, 1995; Toklu et al., 2009). Similarly, potential donors for yield traits, *viz.*, number of pods per plant and weight of the seed were observed in *L. culinaris* ssp. *orientalis* and *L. lamottei.*

## 5 Application of omics-technologies: Landscape of lentil genomic resources, developed lines and genome assemblies

The productivity gains so far achieved in lentils are largely based on the use of traditional breeding approaches. Developing climate-resilient smart crop varieties with broad-spectrum tolerance to withstand multiple simultaneous stresses in a short span of time would not be possible by traditional crop breeding alone. Further, since the economically important crop traits are mostly quantitative in nature and get highly affected by their immediate environment, such GxE interactions add another level of complexity to breeding programs. The deployment of a multitude of advanced genomics tools in integration with traditional breeding pipelines, however, has made this task achievable in many important crop species (Maghuly et al., 2022). These new genomic tools and technologies including molecular DNA markers, cutting-edge sequencing technologies, high-density genotyping and phenotyping platforms, genome mapping, genome dissection, genomic selection, predictions and editing methods have expedited the breeding of improved varieties (Sihag et al., 2021; Kumar et al., 2021; Dhakate et al., 2022). The availability of high quality reference genomes is constantly growing due to the access to newer methods to sequence large whole genomes with affordability. The advancement in allied disciplines of bioinformatics, statistics, data science and modelling strategies coupled with traditional breeding are assisting in realizing enormous sustainable agricultural productivity gains much faster than before. The integration of traditional breeding methods with a new era of molecular breeding can tackle the challenges of changing global climate and sustain the crop productivity for future food and nutritional security (Huang et al., 2022; Yaqoob et al., 2023). Although, limited efforts have gone into the genomics-assisted breeding of lentil so far (Tiwari et al., 2022; Zeroual et al., 2023), an accelerated development of genomic resources during the last decade raises many hopes (Kumar et al., 2015; Kumar et al., 2021 **).**

Lentil CWRs have been extensively studied for useful traits that can serve as important genomic resources for future breeding programs. Various molecular marker systems such as restriction fragment length polymorphisms (RFLPs), inter simple sequence repeats (ISSRs), simple sequence repeats (SSRs), randomly amplified polymorphic DNAs (RAPDs), and amplified fragment length polymorphisms (AFLPs) have been used to study the genetic diversity and phylogenetic relationships within the genus *Lens* (Havey and Muehlbauer, 1989; Abo-elwafa et al., 1995; Fratini et al., 2004; Ferguson et al., 2000; Sharma et al., 1995; Sharma et al., 1996; Fikiru et al., 2007; Babayeva et al., 2009; Hamwieh et al., 2009; Toklu et al., 2009; Gupta et al., 2012a; Gupta et al., 2012b; Kumar et al., 2014; Idrissi et al., 2015; Kushwaha et al., 2015; Mekonnen et al., 2015; Wong et al., 2015; Dissanayake et al., 2020; Hussain et al., 2022 **).**

Many new marker systems like (DAMD- directed amplification of minisatellite), (iPBS-transcriptase primer binding site), sequence-related amplified polymorphism (SRAP) have also been used in assessing genetic diversity and characterization of *Lens* species (Bermejo et al., 2014). Based on all these marker systems, *Lens* species can be readily distinguished from each other and support the earlier reports that *L. culinaris* ssp. *orientalis* is the progenitor species of the cultivated one (Alo et al., 2011; Liber et al., 2021). Among all the above-mentioned DNA molecular markers, simple sequence repeats (SSRs), have been most extensively utilized for the construction of lentil linkage maps (Hamwieh et al., 2005; Verma et al., 2014) and have been coupled with transcriptomic analysis as well (Kaur et al., 2011; Kant et al., 2017).

More recently, the availability of large transcriptomic and genomic data of lentils generated using cutting-edge sequencing have facilitated the generation of high throughput marker systems like expressed sequence tags (ESTs) and single nucleotide polymorphisms (SNPs) that have been extensively used singly or coupled with SSRs for lentil genotyping, genetic diversity, phylogenetics and linkage mapping. (Cheung et al., 2006; Bouck and Vision, 2007). Besides molecular markers, the access to suitable mapping populations are a prerequisite for executing efficient molecular breeding programs. To identify the genomic regions associated with desired crop traits, many RIL mapping populations have been developed in lentil (Tullu et al., 2008; Aldemir et al., 2017; Ma et al., 2020; Gela et al., 2021a). Further, the availability of a reference genome is a prerequisite for modern breeding programs as it allows comparison and identification of allelic variants in different populations, their mapping followed by establishing their connection with phenotypic variation, if any.

Genome sequencing of *Lens* species is challenging as they possess large (approx. 4 Gbp; Arumuganathan and Earle, 1991 **)** and complex genomes. A draft genome of lentil, an exome capture array based on the ‘CDC Redberry’ lentil cultivar was developed using short read transcript resources (Ramsay et al., 2016). The probes were designed to target both cultivated lentil and wild species, and the phylogenetic analyses corroborated previous conclusions of existence of 4 distinct gene pools (Ogutcen et al., 2018). In the cultivar ‘CDC Redberry’ genome assembly was generated covering 3.8 Gbp from genome size of 3.92-Gbp (Ramsay et al., 2021; https://knowpulse.usask.ca/genome-assembly/Lcu.2RBY). A long-read assembly of the lentil cultivar ‘PBA Blitz’ is also completed (Guerra-García et al., 2021). A complete genome assembly is also generated from the related species *L. ervoides* accession ‘IG 72815’ with estimated genome size of 3.4-Gbp (Ramsay et al., 2021, https://knowpulse.usask.ca/genome-assembly/Ler.1DRT) (Guerra-García et al., 2021). Recently, efforts to develop genome assemblies have also been extended to lentil CWRs. Genome assembly (Ramsay et al., 2021) and complete chloroplast genome sequencing of wild *L. ervoides* (Tayşi et al., 2022) and transcriptome assemblies of cultivated lentil and its CWRs (Gutierrez-Gonzalez et al., 2022) are quite encouraging.

The genome and transcriptome assemblies in cultivated lentil and its CWRs will help in going beyond simple genetic maps for dwelling upon the structural rearrangements that have shaped the evolution of genus *Lens* and comparison across legume species to earmark the genetic control of traits of common interest. With all these developments, the genus lentil is picking pace with the omics technologies gradually and steady growth is anticipated in the coming years towards the molecular breeding of this important pulse crop.

## 6 Genetic linkage maps and mapping populations of lentil

The construction of detailed genetic linkage maps is essential for localization of genes and/or QTLs linked to desirable traits, map-based cloning and MAS **(** Semagn et al., 2006 **).** The first lentil genetic linkage map was constructed by Zamir and Ladizinsky (1984) using isozymes and one morphological marker. Subsequently DNA markers based genetic linkage maps have been constructed by many workers (**Table 3** **)** using RFLPs, ISSRs, SSRs RAPDs, AFLPs and SNPs. These maps have been used for localization of genes and QTLs linked to desirable traits, map-based coning and MAS. The first lentil linkage map was constructed using morphological markers and isozymes (Zamir and Ladizinsky, 1984; Havey and Muehlbauer, 1989; Vaillancourt and Slinkard, 1993; Tahir and Muehlbauer, F., 1994 **)** followed by the usage of PCR markers (Eujayl et al., 1998; Rubeena and Taylor, 2003; Hamwieh et al., 2005; Phan et al., 2007; Tullu et al., 2008; Saha et al., 2010a; Verma et al., 2015 **)** and SNPs (Fedoruk et al., 2013;.Gujaria-Verma et al., 2014; Ates et al., 2018; Polanco et al., 2019) The length of these maps varies from 333 centimorgans (cM) to 1868 cM with an average density of 8.9 cM. These maps have been constructed using interspecific crosses involving cultivated lentil and wild species *L. ervoides, L. odomensis* and *L. orientalis)* and RIL populations (Eujayl et al., 1998; Gujaria-Verma et al., 2014; Polanco et al., 2019 **)** and have revealed a direct macro-syntenic relationship between *L. culinaris* ssp. *culinaris* and *Medicago truncatula* genetic maps.

**Table 3:** Genetic linkage maps with QTLs/associated genes.

Population	Species	Markers	QTLs	Traits	Map (cM)	References
RIL	*‘L.culinaris* ssp. *orientalis* × *L. culinaris ssp. culinaris’*	RAPDs, RFLPs, AFLPs, and morphological markers	–	–	1073	Eujayl et al., 1998
F_2_	*‘L. culinaris* ssp. *culinaris* × *L. culinaris* ssp. *orientalis*’	RAPDs, ISSRs, SSRs, AFLPs, CAPS, SRAPs, and morphological markers	–	–	2234	Durán et al., 2004
F_2_	*‘L. culinaris* ssp. *culinaris* × *L. culinaris* ssp. *orientalis*’	RAPDs, SSRs, ISSRs, AFLPs, and morphological markers	23 QTLs	Plant growth habit and plant yield	2172.4	Fratini et al., 2007
RIL	‘ILL5588 × L692-16-1’	SSRs and AFLPs	QTLs	*Fusarium* wilt	751	Hamwieh et al., 2005
F_2_	‘ILL5588 × ILL7537’	RAPDs, ISSRs, and RGAs	–	–	784.1	GorimVandenberg, 2017
F_2_	‘ILL5588 × ILL7537 and ILL7537 × ILL6002’	RAPDs, ISSRs, AFLPs, and morphological markers	5 QTLs	*Ascochyta* blight resistance	412.5	GorimVandenberg, 2017
F_5_	‘ILL5722 x ILL5588’	SSRs and cross genera ITAPs	–	–	928.4	Phan et al., 2007
RIL	‘Cv Eston × PI 320937’	AFLPs, RAPDs, and SSRs	QTLs	Anthracnose resistance	1868	Tullu et al., 2006a
RIL	‘Cv Eston × PI320937’	–	11 QTLs	Earliness and plant height	–	Tullu et al., 2008
RIL	*‘L. culinaris* ‘Eston’ and *L. ervoides* (Brign.) ‘Grande IG 72815’	Morphological markers	–	Anthracnose resistance	–	Tullu et al., 2013
RIL	‘Precoz × WA 8649041’	RAPDs, ISSRs, AFLPs, and morphological markers	–	–	1396	Tanyolac et al., 2010
RIL	‘ILL 6002 × ILL 5888’	RAPDs, SSRs, SRAPs, and morphological markers	Many QTLs	Days to flowering, Seed diameter, plant height	1565	Saha et al., 2013
RIL	‘ILL 6002 × ILL 5888’	RAPDs, SSRs, and SRAPs	1 QTL	*Stemphylium* blight resistance	38.4 to 256.2	Saha et al., 2010a
RIL	‘WA8649090 × Precoz’	RAPDs, ISSRs, and AFLPs	5 QTLs	Cold winter hardiness, leaf area	1192	Kahraman et al., 2004; Kahraman et al., 2010
RIL	‘Northfield (ILL5588) × cv. Digger (ILL5722)’	SSRs, ESTs, and SSRs,	6 QTLs	*Ascochyta lentis* resistance	1156 to 1392	Gupta et al., 2012a
F_2_	‘L830 × ILWL77’ (*L. culinaris* ssp. *culinaris* and *L. culinaris* ssp. *orientalis)*	RAPDs, ISSRs, and SSRs	–	–	3843	Gupta et al., 2012b
RIL	‘CDC Robin × 964a-46’	SNPs	–	–	834.7	Sharpe et al., 2013
RIL	‘CDC Robin × 964a-46’	SSRs, SNPs, and seed colour genes	–	Cotyledon color, seed thickness, seed diameter, plumpness	697	Fedoruk et al., 2013
F_2_	*‘L. culinaris* ssp. *culinaris* × *L. culinaris* ssp. *orientalis’*	RAPDs, SSRs, CAPS and SRAPs	–	*TFL1* gene and other markers	2234.4	de la Puente et al., 2013
F_2_	‘Karcadağ x Silvan’	SSRs markers	–	–	–Andeden et al., 2013
RIL	‘Cassab × ILL 2024’	SSRs and SNPs	–	Boron Tolerance	1178	Kaur et al., 2014
RIL	‘Precoz × WA 8649041’	SNPs	–	–	540	Temel et al., 2014
RIL	‘Precoz × WA8649041’	SSRs, and ISSRs and SNPs	–	–	432.8	Temel et al., 2015
RIL	‘Precoz × L830’	SSRs	2 QTLs	Seed weight and seed size	1183.7	Verma et al., 2015
RIL	‘Precoz × WA8649041’	RAPDs, ISSRs, SSRs and AFLPs	1 QTL	Flowering time	1396.3	Kahraman et al., 2015
RIL	‘ILL 8006 × CDC Milestone’	SSR, AFLP, and SNPs	21 QTLs	Iron concentration in seeds	497.1	Aldemir et al., 2017
RIL	‘PI 320937 × Eston’	SNPs and SSRs	4 QTLs	Selenium uptake	4060.6	Ates et al., 2016
RIL	Indianhead×Northfield;, Indianhead×Digger; Northfield×Digger	SNPs, SSRs and EST-SSRs	QTLs	*Ascochyta* blight resistance	1461.6, 1302.5 and 1914.1	Sudheesh et al., 2016
RIL	‘ILL6002×ILL5888’	SNPs and SRAPs	–	Drought tolerance related root and shoot traits	–	Idrissi et al., 2016
RILs	“CDC Redberry” x “ILL7502”	DArTs	6 QTLs	Manganese uptake	977.47	Ates et al., 2018
RIL	‘ILL2024×ILL6788’	SNPs and SSRs	1 QTL	Boron tolerance	1057	Rodda et al., 2018
RIL	*‘L. culinaris* cv. Alpo × *L. odemensis* accession ILWL235’	SNPs	10 QTLs	Agronomic traits	5782.19	Polanco et al., 2019
RIL	‘WA8649090 x Precoz’	RAPDs, SSRs and ISSRs	6 QTLs	Early Plant vigour	809.4	Mane et al., 2020
F_2_	‘L-4147 × PDL-1’	SSRs	–	Salinity stress tolerance at seedling stage	133.02	Singh et al., 2020
RIL	*‘L. culinaris* cv. Lupa and *L. orientalis* BGE 016880’	SNPs	13 QTLs	Flowering time	5923.3	Yuan et al., 2021
RIL	*‘L. culinaris* cv. Eston × *L. ervoides* cv. IG 72815’	SNPs	2 QTLs	Anthracnose resistance	3252.8	Gela et al., 2021b
RIL	‘ILWL 180 *(L. orientalis*) × ILL 6002(*L. culinaris*)’	SNPs	QTLs and candidate genes	*Ascochyta* blight resistance	545.4	Dadu et al., 2021

The first extensive genetic linkage map of lentil with molecular markers was constructed by Eujayl et al. (1998) saturated with total 177 markers comprised of morphological and molecular (RAPD, RFLP, and AFLP) markers using 86 RILs generated from an interspecific cross. Rubeena and Taylor, (2003) generated a lentil genetic map with 9 linkage groups (length 784.1cM) saturated with 3 RGA, 100 RAPD and 11 ISSR markers using a F_2_ population developed from a cross of cultivars differing in resistance to *Ascochyta* blight. Likewise, Hamwieh et al. (2005) constructed a map using 283 markers linked to *Fusarium* wilt disease.

An F_5_ population of *L. culinaris* ssp. *culinaris* was used to construct a gene-based genetic linkage map (928.4 cM long) with 7 linkage groups utilising 18 SSR and a high number of intron-targeted amplified polymorphic (79 ITAP) markers (Phan et al., 2007). The linkage groups detected in the above study comprised of 5–25 markers with 80.2 to 274.6 cM length variations. A direct macro-syntenic relationship between *L. culinaris* ssp. *culinaris* and *Medicago truncatula* genetic maps was revealed by analysing mapped markers previously assigned to the *M. truncatula* genetic and physical maps. Tullu et al. (2008) developed a lentil map (1868 cM long) for earliness and plant height traits using 207 markers (AFLPs, RAPDs and SSRs), and revealed 12 linkage groups with an average marker density of 8.9 cM. A molecular linkage map of 1396.3 cM length with 11 linkage groups was constructed using 166 markers (morphological, RAPDs, ISSRs and AFLPs) in an RIL population (Tanyolac et al., 2010). A subset (420) of SNPs were also selected for amplification and mapping in the F_7_ RIL population (Precoz × WA8649041) along with 15 SSR, and 29 ISSR markers.

Interspecific populations were raised using wild and cultivated taxa (*L. culinaris* and *L. orientalis*, *L. odemensis* and *L. ervoides*) for the purpose of constructing genetic maps (Eujayl et al., 1998; Durán et al., 2004; Gujaria-verma et al., 2014; Polanco et al., 2019). An F_2_ segregating intersubspecific population (*L. culinaris* ssp. *culinaris* and *L. culinaris* ssp. *orientalis*), using 235 markers (SSR, ISSR and RAPD) was mapped covering 3843.4 cM into 11 linkage groups (LGs), with an average marker distance of 19.3 cM (Gupta et al., 2012a). A previous *Lens* genetic map representing *L. culinaris* ssp. *culinaris* × *L. culinaris* ssp. *orientalis* was improved by adding 31 new markers, reaching upto 190 markers that formed eight linkage groups covering 2234.4 cM (de la Puente et al., 2013). Andeden et al. (2013) constructed a linkage map using F_2_ population of the cross between Karcadağ x Silvan cultivars using 47 SSR markers with 43 loci assigned to six linkage groups. A consensus linkage map (977.47 cM long), has been made using diversity arrays technology (DArT) markers with 3 RIL mapping population including ‘ILL8006’ x ‘CDC Milestone’, ‘PI320937’ x ‘Eston’ and ‘CDC Redberry’ x ‘ILL7502’ (Ates et al., 2018). It covered a total of 9,793 markers with an average distance of 0.10 cM in between the markers. With seven linkage groups the length of the map was comparable with that of Sharpe et al. (2013).

Many lentil mapping populations have been raised using intra- and interspecific crosses between such as drought sensitive ‘JL-3’ and drought resistant ‘PDL-1’ and ‘FLIP-96-51’ cultivars, in order to study the inheritance mechanism of drought tolerance and identify the linked polymorphic markers. Bulk segregant analysis results have shown the association of seven out of 51 SSR markers with drought tolerance detected at the seedling stage (Singh et al., 2016). These seven markers were screened and mapped (133.2 cM distance) in F_2_ mapping population (JL-3×PDL-1) of 101 individuals. As evident, lentil linkage map studies have benefitted a lot by application of SSR markers.

SNP markers have also been extensively utilized in lentil and have contributed enormously to linkage mapping, genetic diversity and trait association studies (Kaur et al., 2011 **;** Gujaria-Verma et al., 2014; García-García et al., 2019; Pavan et al., 2019; Wang et al., 2020). Many studies have used SNP markers to identify genetic markers associated with drought tolerance and devlop high-resolution maps. About 377 SNPs were identified from TOG sequences in *L. ervoides* and used to generate a map with seven linkage groups (Gujaria-Verma et al., 2014). In another study, Gupta et al. (2012b) used among other markers a set of 15 *M. truncatula* EST-SSRs in an RIL population of ‘Northfield (ILL5588) × cv. Digger (ILL5722)’ which clustered across 1156.4 cM map length into 11 linkage groups. A genetic linkage map of 697 cM was developed in *Lens* using 563 SNPs, 10 SSRs, and four loci of seed color (Fedoruk et al., 2013). Another recent technique, genotyping by sequencing (GBS) approach was used in the genus *Lens* to generate a total of 266,356 SNPs across whole genome for use in phylogenetic and population structure analysis (Wong et al., 2015). A comprehensive characterization of SNPs has been achieved in *L. culinaris* and wild *L. ervoides* genotypes (Khazaei et al., 2016). Recently, GBS-based Diversity array technology (DArT) markers were used in lentil for the identification of SNPs and development of high-resolution genetic maps **(** Pavan et al., 2019; Dadu et al., 2021 **).** However, despite above efforts, MAS has not been widely used in lentil breeding due to poor association of markers with the desired genes and the poor resolution issues associated with genetic maps.

Nevertheless, the availability of these genetic linkage maps, along with the draft genome assemblies and high-throughput marker systems, has greatly facilitated the genomics-assisted breeding of lentil for the development of climate-resilient smart crop varieties with broad-spectrum tolerance to withstand multiple simultaneous stresses.

## 7 QTL and association mapping

The rapid development of an array of molecular markers in the past few decades has enabled the identification of many useful QTLs linked to agronomic traits in many crops. QTL mapping is based on linkage mapping and genotypic data and has been utilized for marker-trait association or marker-assisted breeding in many crops including lentil.

Genetic mapping studies have helped in identifying many genes and QTLs controlling abiotic and biotic stress tolerance, growth, development and nutritional parameters have been mapped in lentil (Eujayl et al., 1998; Tullu et al., 2003; Tullu et al., 2006b; Durán et al., 2004; Kahraman et al., 2004; Hamwieh et al., 2005; Gupta et al., 2012a; Saha et al., 2013; Kaur et al., 2014; Ates et al., 2016; Idrissi et al., 2016; Sudheesh et al., 2016; Rodda et al., 2017; Ates et al., 2018; Polanco et al., 2019; Ma et al., 2020; Mane et al., 2020; Gela et al., 2021a, b). The details about genetic linkage maps constructed with QTLs governing the traits of interest have been listed in **Table 3**. Lately, mapping of quantitative traits like mineral concentration in seeds, days to flower, desirable seed characters and *Aphanomyces* root rot has been carried out by association mapping **(** Khazaei et al., 2017; Khazaei et al., 2018; Neupane, 2019; Ma et al., 2020 **).**

The flowering time and seed characteristics are important productivity-related crop traits. In this regard, five QTLs each for the height of first ramification and flowering time, seven for pod dehiscence, three for plant height, and one each for number of shoot and seed diameter were detected in inter-subspecific genetic map in *Lens* (Durán et al., 2004). Many QTLs for plant height and earliness were identified from RILs using cross between ‘Eston × PI320937’ (Tullu et al., 2008). RILs derived from a cross between genotypes ‘WA 8649090 × Precoz’ were used to detect QTLs for winter survival and injury (Kahraman et al., 2004). For seed diameter and weight, three and five QTLs respectively were identified (Saha et al., 2013). Further, in 78 RIL populations derived from a cross between a cultivar ‘Alpo’ of *L. culinaris* and *L. odemensis* accession ‘ILWL235’, three QTLs for seed size and one each QTL for stem pigmentation, spotting on the seed coat, the color of flower and timing of flowering were identified. QTLs for the seed weight and seed size traits were identified in an RIL derived from cross between *L. culinaris* cultivars ‘Precoz x L830’ which generated one QTL each for the traits (seed weight and size) present on the same linkage group (Verma et al., 2014).

Among the biotic stresses, *Ascochyta* blight*, Stemphylium* blight, anthracnose and rust diseases represent the most potent pathogens that limit lentil productivity worldwide. Many QTLs associated with these pathogens have been identified. These genomic resources can be extremely helpful in lentil breeding for biotic resistance and productivity gains. RIL population developed from a cross between *L. culinaris* ‘Eston’ and ‘PI 320937’ was used to identify markers associated with *Ascochyta* blight resistance, using a QTL analysis (Tullu et al., 2003; Tullu et al., 2006b). Further, three more QTLs were detected for *Ascochyta* blight resistance at seedling and pod maturity stages against *Ascochyta lentis* (Gupta et al., 2012a). Similarly, Sudheesh et al. (2016) identified multiple QTLs associated with *A. lentis* in 112 and 117 RILs obtained between crosses ‘IH (Indian Head) x DIG (Digger)’ and ‘IH x NF (Northfield)’, respectively. In yet another F_2_ population derived from ‘ILL7537 × ILL6002’, three QTLs accounting for 47% (QTL-1 and QTL-2) and 10% (QTL-3) of *Ascochyta* blight resistance variation were mapped. Further, QTLs conferring resistance to *Stemphylium* blight and rust diseases (caused by *Uromyces vicia-fabae*) using RIL populations were also identified (Saha et al., 2010a; Saha et al., 2010b). The RIL population for *Stemphylium* blight resistance (‘ILL5888 × ILL-6002’), showing contrasting agro-morphological traits, were used to detect three QTLs related to days to 50% flowering. Composite interval mapping from an RIL population (F_9_) between two *L. ervoides* accessions, revealed 11 QTLs with associated resistance to *Colletotrichum lentis* resistance at different stages against anthracnose, and three QTLs for *Stemphylium botryosum* resistance against blight disease (Bhadauria et al., 2017). LAB C01 resistance at BC2F3:4 generation was screened for the race 0 of anthracnose (*C. lentis*) and *Stemphylium* blight (*S. botryosum*) and identified QTLs on chromosomes 3 and 7 (Gela et al., 2021b). 15 putative genes associated with resistance to *Aphanomyces* root rot (Ma et al., 2020) have been identified on seven QTL clusters using QTL and association mapping. Differential expression of three of these genes at the early stages of infection was correlated with ARR resistance (Ma et al., 2020).

Climate change-inflicted abiotic stresses have affected yield and lentil productivity substantially, hence, identification of genomic resources can be really helpful in developing stress-tolerant varieties. In an RIL population of a cross between lentil accessions ‘ILL6002 and ILLL5888’, Idrissi et al. (2016) identified eighteen QTLs with different root and shoot traits under drought stress. Sodicity represents one of the most important abiotic stresses responsible for reduction in crop yields. By crossing lentil salt-sensitive ‘L-4076 and L-4147’ and salt-tolerant genotypes ‘PDL-1 and PSL-9’, Singh et al. (2020) identified a QTL linked to seedling survival under salinity conditions. Further, efforts to link a QTL to cold hardiness have resulted in the identification of a stable QTL, that expressed uniformly in different cold conditions. This QTL can be pipelined for MAS **(** Kahraman et al., 2004 **).** Although the above reports highlight the usage of lentil genotypes harboring the stress-tolerant QTLs, efforts must be extended to CWRs to explore more useful genomic resources which can be used in appropriate breeding strategies to improve lentil productivity.

Plant growth depends on many factors and alterations in minerals and/or micronutrient uptake plays a key role in determining plant growth in changing climate scenarios. Studies on mineral ion uptake in lentils identified a few QTLs linked to boron, selenium, manganese and other ions uptake (Kaur et al., 2014; Ates et al., 2016; Khazaei et al., 2017; Ates et al., 2018; Khazaei et al., 2018 **).** Further studies in this direction can lead to breeding of biofortified micronutrients rich lentil.

For realizing the full potential and applications of identification of QTLs and other genomic resources in the lentil improvement, association and mapping studies are extremely important so that these resources can be effectively utilized in MAS. Some useful attempts have been made in this direction. For instance, Kaur et al. (2014) identified QTLs in ‘Cassab × ILL2024’ mapping population related to boron tolerance. The authors used transcriptome sequencing generated SNPs and EST-SSRs for simple interval mapping (SIM) and composite interval mapping (CIM). A comparison of the flanking markers to genome sequences with model species like *M. truncatula* could identify many candidate genes associated with micronutrient (Boron) tolerance that might become useful in marker assisted breeding. Similarly, Fedoruk et al. (2013) used SNPs, SSRs and seed coat color markers in RIL population of lentil to identify QTLs for seed dimension. Significant QTLs on 6 linkage groups were identified like linkage group 2 with seed coat color pattern and linkage group 1 with cotyledon color locus (Fedoruk et al., 2013). Polanco et al. (2019) analysed F_7_ RILs (*L. culinaris x L. odemensis*) and identified a single QTL controlling ‘time to flowering’ and three QTLs for ‘seed size regulation’. QTLs were also mapped in lentil for *Ascochyta* blight resistance in chromosome 6. Further, Neupane (2019) observed 4 QTLs for ‘days to flowering’ after evaluating 324 lentil accessions in multiple locations in different parts of the world. The mapping population was a cross between accessions ‘IPL 220 and ILWL 118’ of wild species *L. orientalis* (Kumar et al., 2019). A QTL hotspot was observed consisting of six QTLs for lengths of root, shoot and seedling within a map distances of 56.61-86.81 cM range on LG1 using F_10_ RIL population of cross ‘WA8649090 x Precoz’ (Mane et al., 2020). Likewise, a total of 143 accessions were analysed by GWAS to establish associations between prebiotic carbohydrates and candidate genes (Johnson et al., 2021). The study identified many SNPs and associated genes controlling useful traits. This study can further guide the molecular breeding programs based on prebiotic carbohydrates in lentil.

In summary, many studies have used transcriptome profiling and QTL mapping to identify genes and genic regions associated with abiotic and biotic stress tolerance, growth, development and nutritional parameters in lentils. The studies have involved use of RIL populations and various methods such as transcriptome sequencing, SNPs, EST-SSRs, SSRs, seed coat color markers, GWAS and more. The studies have identified a wide range of QTLs associated with boron tolerance, proline metabolism, membrane proteins, defense-related functions, and phytohormones, as well as QTLs for traits such as plant height, flowering time, seed characteristics, time to flowering, cold hardiness, *Ascochyta* and *Stemphylium* blight resistance, rust resistance, salinity and drought tolerance. These findings have important implications for marker-assisted breeding and the development of more stress-tolerant lentil cultivars.

## 8 Transcriptomic profiling to dissect the functionality of abiotic and biotic stresses

Transcriptomic studies provide information about functionality and regulation of genes and show how reprogramming at transcriptional level can modulate innate physiological parameters in plants to withstand external stresses. Transcriptomic studies in lentil have resulted in identification of many candidate genes/loci linked to useful agronomic traits **(** Kaur et al., 2011; Sudheesh et al., 2016; Cao et al., 2019; García-García et al., 2019; Morgil et al., 2019; Singh et al., 2019; Wang et al., 2020; Dadu et al., 2021; Kumar et al., 2021; Tiwari et al., 2022 **).** ESTs-based methods coupled with NGS are widely used for transcriptome studies. In lentil, 33,371 ESTs are currently publicly available (Kumar et al., 2021). A high quality of 847,824 sequence reads and 84,074 unigenes transcriptome assemblies were generated as a result of massive transcriptome sequencing in lentil (Sharpe et al., 2013; Verma et al., 2013). Further, an EST library was developed using lentil cultivars with varying seed phenotypes by Vijayan et al. (2009), while Kaur et al. (2011) revealed 2,393 loci for EST-SST markers upon cDNA sequencing of six lentil genotypes. Interestingly, 47.5% polymorphism was revealed among 13 different lentil genotypes screened with 192 out of these markers. Immediately after, a large number of ESTs were generated using tissues of leaves infected with *C. truncatum* in lentil (Bhadauria et al., 2011; Kumar et al., 2014).

Many studies have tried to unravel the mode of action of various biotic and abiotic stresses with the help of transcriptome profiling in lentil. To study the transcriptome profiling during cold stress, Barrios et al. (2017), performed a Deep Super-SAGE transcriptome analysis on RIL populations of a cross between ‘cold tolerant WA8649041 and susceptible genotype Precoz’ to identify around 300 differentially expressed tags mainly associated with expressing proline rich, dormancy related membrane proteins.

Similarly, to understand the functionality of drought stress response, Singh et al. (2017b) revealed that 11,435 transcripts were up- and 6,934 were down-regulated to study the effect of drought stress in a resistant (PDL-2) and sensitive (JL-3) cultivar in comparison with the control. Further, DEG (Differentially expressed gene) analysis showed upregulation of genes involved in electron transport chain, glucose metabolism, TCA cycle and down regulation of photosynthetic functions and photorespiration in the tolerant cultivar **(** Singh et al., 2017a; Morgil et al., 2019 **).** The latter study further showed that the number of DEGs in roots of *L. culinaris* cultivar ‘Sultan’ increased from 2,915 to 18,237 in short-term and long-term drought conditions, respectively **(** Morgil et al., 2019 **).** A similar transcriptomic profiling has been done by Singh et al. (2019) to study the mechanism of heat stress tolerance. Heat stress is one of the major abiotic challenges for reduced crop production under changing climate scenarios. By comparing the heat tolerant lentil cultivar ‘PDL-2’ with heat sensitive ‘JL-3’ cultivar, Singh et al. (2019) could identify as many as 16,817 heat responsive DEGs, with their number being higher in heat tolerant cultivar. Functionally, the observed DEGS were mostly correlated with secondary metabolism, wax deposition, cell wall deposition enzymes and many transcription factors (Singh et al., 2019 **).** A transcriptome annotation with 26,449 EST-SSR markers in six lentil genotypes followed by a selection of 276 screened markers to circumscribe 94 accessions showed 125 markers to be polymorphic among the analysed accessions (Wang et al., 2020 **)**

The biotic stresses in the form of *Ascochyta* and *Stemphylium* blights, anthracnose and rust contribute to major losses ranging upto 70% in lentil production across the world (Singh et al., 2017a; Cao et al., 2019). The transcriptomic studies (Cao et al., 2019; Singh et al., 2019; Mishra et al., 2021; Tiwari et al., 2022) have largely focussed on foliar diseases caused by the two most potent lentil pathogens *A. lentils* and *S. botryosum*. The transcriptome profile was studied in two *L. ervoides* cultivars ‘LR-66-637’ (resistant) and ‘LR-66-577’ (susceptible) to *S. botryosum*. A total of 8,810 disease responsive genes along with 1,284 DEGs were identified and as many as 712 genes were upregulated in resistant cultivar as compared to 572 in the susceptible one (Cao et al., 2019). Similarly, Khorramdelazad et al. (2019), studied the transcriptome profiling of ‘ILL7537’ (resistant) and ‘ILL6002’ (susceptible) lentil cultivars infected with *A. lenti*, after 2, 6 and 24 hours after the infection to reveal upregulation of two genes involved in defense-related functions namely calmodulin domain protein kinase-like (*CDPK*) genes, and LRR-receptor like kinase (*LRR-RLKs*) (Khorramdelazad et al., 2019 **).** Interestingly, some common DEGs expressed during infection with both the above pathogens correlated with genes associated with phytohormones, E3 ubiquitin protein, LRR-RLKs, CDPK indicate the prevalence of a common defence mechanism against both these lentil pathogens (Tiwari et al., 2022).

In nutshell, transcriptomic studies in lentils have been widely used to understand the mechanisms of biotic and abiotic stress tolerance and have resulted in identification of many candidate genes and loci linked to useful agronomic traits. The studies have revealed the up- and down regulation of genes involved in different processes such as proline rich dormancy-related and membrane proteins, electron transport chain, glucose metabolism, TCA cycle, photosynthetic functions, photorespiration, and secondary metabolism during cold, drought and heat stress in lentils. Many studies have identified DEGs associated with stress tolerance responses. In addition, transcriptomic studies have been conducted to understand the resistance mechanism to foliar diseases caused by pathogens such as *Ascochyta* and *Stemiphylium* and have revealed the upregulation of defense-related genes such as calmodulin domain protein kinase-like (CDPK) and LRR-receptor like kinase (LRR-RLK) in resistant cultivars. Overall, these studies have provided valuable insights into the molecular mechanisms of stress tolerance and resistance in lentils and have potential applications in breeding programs aimed at improving the crop’s stress tolerance and disease resistance.

## 9 Phenomics, Proteomics and Metabolomics: Recent emerging areas in modern breeding of lentil

The large-scale genomics datasets can result in practical applications once they are correlated with the phenotypes or the phenome (Mir et al., 2019). The conventional manual phenotypic approaches are lately getting replaced by through-put sensor-based phenotypic methods that use ‘artificial intelligence’ and ‘machine learning’ approaches to increase precision and speed of phenotyping (Singh et al., 2016; Tiwari et al., 2022). For example, a comparison of conventional phenotyping with high throughput (HTP) digital red-green-blue (RGB) imaging followed by fluorescence scanning revealed that the latter method had better precision and consistency (Dissanayake et al., 2020). Proteomics studies involving translational and post-translational studies on peptides and proteins, once the candidate genes and loci get identified by genomics studies are important parts of the larger process of crop trait improvement. Likewise, metabolomics signifies the culmination of all the aforementioned genomics technologies and shows a direct correlation with the phenotypes. Researchers have begun to look into the drought and salinity stress management by analysing contrasting lentil genotypes **(** Scippa et al., 2008; Caprioli et al., 2010; Scippa et al., 2010; Muscolo et al., 2015; Skliros et al., 2018; Shaheen et al., 2022 **)**, although more research is needed in this area.

Recent efforts have tried to identify genomic regions that are associated with markers and traits in lentils. For instance, Tiwari et al. (2022) found 19 common metabolites in lentils that belong to phenolic and organic acids, saccharides, and flavan/flavanol and flavaone derivatives. This study suggests that there is a dynamic cross-talk during stress management in plant systems, and it highlights the need for comprehensive integrated future investigations in lentil and other crop species. It is important to identify pan-stress-ameliorating genes and/or loci and common stress mitigation pathways, if any, as in the natural field conditions crops are exposed to multiple simultaneous biotic and abiotic stresses. This will be very useful for external stress management and will help to ease the pressure off the agricultural productivity issues. Additionally, the identification of signature peptides and metabolites as markers associated with useful agronomic traits will be helpful in lentil breeding.

## 10 Conclusions and future prospects

Understanding the evolutionary and domestication processes in crop species requires knowledge about the genetic and phenotypic characteristics of available genetic resources such as accessions, landraces and genotypes as well as understanding the genetic basis of divergence. The documented variability serves as the foundation of all crop improvement programs aimed at increasing productivity, disease resistance, stress mitigation and climatic adaptations. The genetic and genomic analysis of crop wild resources (CWRs) across cereals, legumes, oils and other diverse groups of plants has demonstrated that the CWRs possess high heterozygosity and many useful crop traits that can be used in crop breeding programs. The availability of enormous CWRs and land races offers interesting opportunities for wild gene introgression into the cultivated gene pools of legumes and other crop species.

The last few decades have seen an unprecedented growth in the development of methods for genetic research and breeding in plants. Plant breeding exercises have advanced greatly from the usage of a plethora of molecular markers to next generation sequencing to genotyping-by-sequencing. At the same time, assembly of large and complex genomes, development of high-density genetic maps for high resolution QTL mapping, genome-wide association studies, development of genomic resources in the form of mini and/or core populations, trait-specific mapping populations, multi-parent advanced generation inter-cross (MAGIC) and nested association mapping (NAM) populations, and the development of pan or super-pan genomes of cultivated species and CWRs through whole genome sequencing (WGS) have substantially modernized the crop breeding programs. These technologies have enabled the identification and characterization of genes associated with important agronomic traits such as disease resistance, drought tolerance and yield, which can be used to develop new cultivars with improved traits.

The legume agricultural production system including lentils has inherently been constrained by cultivation in limited geographical habitats, poorly defined breeding histories, genetic bottleneck and erosion, intensive agricultural systems and novel pathogens under global climatic changes. Since the genetic diversity locked in CWRs is considered to offer viable solutions to food productivity problems, intensive efforts should be undertaken to collect, characterize and protect CWRs of grain legumes. Recently, a shift has been noted during the germplasm characterization exercises towards cataloguing diversity at the desirable gene level rather than the phenotype level. Furthermore, since the characterization of genetic diversity and dissection of complex traits are pivotal to the idea of genetic improvement, a centralized data base management system should be put in place to host the collated information about the wild alleles controlling specific traits.

Overall, in the small genus *Lens*, an important plant-based protein source, which was once considered an orphan species, significant wild germplasm characterization efforts have taken place. These efforts have led to advancements in understanding the genomic relationships between the wild and cultivated lentil genomes, identification of genes, QTLs and traits associated with desired crop traits and stress management, and the development of genetic maps and databases, global genotyping, the use of marker assisted and genomic selection techniques, draft genomes’ assemblies, complete chloroplast genome sequencing and transcriptome assemblies of cultivated lentil and its CWRs (Gutierrez-Gonzalez et al., 2022). These developments have assisted in unravelling the intricacies of genome architecture and the landscape of variability available in the gene pools of cultivated lentil and its wild relatives and evolutionary and domestication history of the species. However, there is still a need for better management of various biotic and abiotic stresses associated with the global climatic changes and to maintain the desired productivity levels for the future food security. One key area of focus is to characterize lentil germplasm resources in their centres of origin, where they are most diverse, in order to identify genes and traits that can help mitigate the effects of climate change and maintain productivity levels for future food security (Chen et al., 2017; Singh et al., 2018). Additionally, it is important to characterize the genetic and phenotypic diversity at individual accession level rather than just at the genotype level that represents a pool of accessions. To achieve genetically enhanced and biofortified lentil, data should be integrated from multiple omics technologies, such as robust marker association studies, machine and AI-assisted phenomics studies, advanced proteomics and metabolomics and biofortification studies carried out in CWRs and the cultivated lentil (Tiwari et al., 2022). All these findings should be represented in a centralized curated data base repository for information sharing to aid future breeding efforts.

The development and use of MAGIC populations has been quite beneficial for gene mapping and function analysis, detection of QTLs, dissection of stress and yield related traits and genetic resource development in the form of elite breeding near isogenic lines (NILs) and recombinant inbred lines (RILs) in legumes such as chickpea, faba bean, pigeonpea, cowpea, soybean and groundnut. These important genomic resources’ development needs attention of lentil breeders.

Most genomic and transcriptomic studies in the genus *Lens* have involved commercial accessions of *L. culinaris*. Recent efforts to develop genome assemblies of *L. culinaris* and wild *L.ervoides* (Ramsay et al., 2021), complete chloroplast genome sequencing of wild *L. ervoides* (Tayşi et al., 2022) and transcriptome assemblies of cultivated lentil and its CWRs (Gutierrez-Gonzalez et al., 2022) are quite encouraging and will help researchers better understand the genomic relationships between wild and cultivated lentil genomes to tap into the unexploited variability lying hidden in CWRs. Many specific legume databases such as Pulse crop data base (https://www.pulsedb.org/), Legume information system (LIS; https://legumeinfo.org; Dash et al., 2016) and KnowPulse (https://knowpulse.usask.ca) are really helpful for accessing useful genetic data for lentil breeding. The future efforts should aim at comprehensive linking of genetic datasets to phenotypes and also connecting these data pipelines under the umbrella of a centralized curated database management system. The implementation of dedicated large scale global legume improvement projects like EVOLVES (https://knowpulse.usask.ca/study/2691111) and European Union’s Horizon 2020 research and innovation program INCREASE (https://www.pulsesincrease.eu/crops/lentil) (Guerra-García et al., 2021) are important strategic policy decisions that will help in the conservation and sustainable use of crop agro-biodiversity in pulse crop species including lentil. These projects provide a way forward to consolidate global efforts in addressing the challenges of climate change.

## Author contributions

Conceptualization VR and AS, Literature survey and Original Draft writing: AS and VR. Tables AS. Review and Editing: VR, AS, RK, RT, MK, AP, MH, SR. All authors have read and approved the MS in the present form. All authors contributed to the article and approved the submitted version.

## References

[B2] Abbo S. Ladizinsky G. (1991). Anatomical aspects of hybrid embryo abortion in the genus*Lens* l. Bot. Gaz 152, 316–320. doi: 10.1086/337895

[B3] Abbo S. Ladizinsky G. (1994). Genetical aspects of hybrid embryo abortion in the *Lens* l. Heredity 72, 193–200. doi: 10.1038/hdy.1994.26

[B4] Abbo S. Ladizinsky G. Weeden N. F. (1992). Genetic analysis and linkage studies of seed weight in lentil. Euphytica 58, 259–266. doi: 10.1007/BF00025258

[B1] Abo-elwafa A. Murai K. Shimada T. (1995). Intra-specific and interspecific variations in *Lens*, revealed by RAPD markers. Theor. Appl. Genet. 90, 335–340. doi: 10.1007/BF0022974 24173922

[B5] Affoh R. Zheng H. Dangui K. Dissani B. M. (2022). The impact of climate variability and change on food security in sub-saharan Africa: Perspective from panel data analysis. Sustainability 14, 759. doi: 10.3390/su14020759

[B6] Ahmad M. McNeil D. L. Sedcole J. R. (1997). Phylogenetic relationships in *Lens* species and their interspecific hybrids as measured by morphological characters. Euphytica 94, 101–111. doi: 10.1023/A:1002960130906

[B7] Ait-El-Mokhtar M. Boutasknit A. Ben-Laouane R. Anli M. El Amerany F. Toubali S.. (2022). “Vulnerability of oasis agriculture to climate change in Morocco,” in Research anthology on environmental and societal impacts of climate change. Eds. Karmaoui A. Barric K. Baig M. B. (Hershey, PV, USA: IGI Global), 1195–1219.

[B8] Aldemir S. Ateş D. Temel H. Y. Yağmur B. Alsaleh A. Kahriman A.. (2017). QTLs for iron concentration in seeds of the cultivated lentil (*Lens culinaris* medik.) *via* genotyping by sequencing. Turk J. Agric. For 41, 243–255. doi: 10.3906/tar-1610-33

[B9] Alo F. Furman B. J. Akhunov E. Dvorak J. Gepts P. (2011). Leveraging genomic resources of model species for the assessment of diversity and phylogeny in wild and domesticated lentil. J. Hered. 102, 315–329. doi: 10.1093/jhered/esr015 21454287

[B10] Alvarez M. T. García P. Pérez de la Vega M. (1997). RAPD polymorphism in Spanish lentil landraces and cultivars. J. Genet. Breed. 51, 91–96.

[B11] Andeden E. E. Derya M. Baloch F. S. Kilian B. Ozkan H. (2013). Development of SSR markers in lentil. in ‘Proceedings of plant and animal genome conference’ XXI P0351 (San Diego, CA).

[B12] Arumuganathan K. Earle E. D. (1991). Nuclear DNA content of some important plant species. Molec Biol. Rep. 9, 208–221. doi: 10.1007/BF02672069

[B13] Asghar M. J. Abbas G. Shah T. M. Atta B. M. (2010). Study of genetic diversity in some local and exotic lentil (*L.* culinaris medik.) genotypes. Pak. J. Bot. 42, 2681–2690.

[B15] Ates D. Aldemir S. Alsaleh A. Erdogmus S. Nemli S. Kahriman A.. (2018). A consensus linkage map of lentil based on DArT markers from three RIL mapping populations. PloS One 13, e0191375. doi: 10.1371/journal.pone.0191375 29351563 PMC5774769

[B14] Ates D. Sever T. Aldemir S. Yagmur B. Temel H. Y. Kaya H. B.. (2016). Identification QTLs controlling genes for Se uptake in lentil seeds. PloS One 11, e0149210. doi: 10.1371/journal.pone.0154054 26978666 PMC4792374

[B16] Babayeva S. Akparov Z. Abbasov M. Mammadov A. Zaifizadeh M. Street K. (2009). Diversity analysis of central Asia and Caucasian lentil (*L.* culinaris medik.) germplasm using SSR fingerprinting. Genet. Resour. Crop Evol. 56, 293–298. doi: 10.1007/s10722-009-9414-6

[B17] Bahl P. N. Lal S. Sharma B. M. (1993). “An overview of the production and problems in southeast Asia,” in In lentil in south asia. proceedings of the seminar on lentils in south Asia. Eds. Erskine W. Saxena M. C. (ICARDA), 1–10

[B18] Bamberg J. B. Hanneman R. E. (2003). Calcium rich potatoes: it’s in their genes. In: Agricultural research magazine (Accessed March 2003).

[B19] Barrios A. Caminero C. García P. Krezdorn N. Hoffmeier K. Winter P.. (2017). Deep super-SAGE transcriptomic analysis of cold acclimation in lentil (*Lens culinaris* medik.). BMC Plant Biol. 17, 111. doi: 10.1186/s12870-017-1057-8 28666411 PMC5493078

[B20] Bayaa B. Erskine W. Abbas A. (1994). Evaluating different methods for screening lentil germplasm for resistance to lentil wilt caused by *Fusarium oxysporum* f. sp. lentis. Arab. J. Plant Prot. 12, 83–91.

[B21] Bayaa B. Erskine W. Hamdi A. (1995). Evaluation of a wild lentil collection for resistance to vascular wilt. Genet. Resour. Crop Evol. 42, 231–235. doi: 10.1007/BF02431257

[B22] Bellard C. Bertelsmeier C. Leadley P. Thuiller W. Courchamp F. (2012). Impacts of climate change on the future of biodiversity. Ecol. Lett. 15, 365–377. doi: 10.1111/j.1461-0248.2011.01736.x 22257223 PMC3880584

[B23] Bermejo C. Gatti I. Caballero N. Cravero V. Martin E. Cointry E. (2014). Study of diversity in a set of lentil RILs using morphological and molecular markers. Aust. J. Crop Sci. 8, 689–696.

[B25] Bhadauria V. Banniza S. Vandenberg A. Selvaraj G. Wei Y. (2011). EST mining identifies proteins putatively secreted by the anthracnose pathogen colletotrichum truncatum. BMC Genomics 12, 327. doi: 10.1186/1471-2164-12-327 21699715 PMC3149586

[B24] Bhadauria V. Ramsay L. Bett K. E. Banniza S. (2017). QTL mapping reveals genetic determinants of fungal disease resistance in the wild lentil species *Lens ervoides* . Sci. Rep. 7, 3231–3240. doi: 10.1038/s41598-017-03463-9 28607439 PMC5468239

[B26] Bouck A. M. Y. Vision T. (2007). The molecular ecologist’s guide to expressed sequence tags. Mol. Ecol. 16, 907–924. doi: 10.1111/j.1365-294X.2006.03195.x 17305850

[B27] Brar D. S. Khush G. S. (1997). “Alien introgression in rice,” in Oryza: from molecule to plant. Eds. Sasaki T. Moore G G. (Dordrecht: Springer), 35–47. doi: 10.1007/978-94-011-5794-0_4 9291958

[B28] Brown A. H. D. (1989). Core collections: A practical approach to genetic resources management. Genome 31, 818–824. doi: 10.1139/g89-144

[B29] Buchwaldt L. Anderson K. L. Morrall R. A. A. Gossen B. D. Bernier C. C. (2004). Identification of lentil germplasm resistant to *Colletotrichum truncatum* and characterization of two pathogen races. Phytopathology 94, 236–249. doi: 10.1094/PHYTO.2004.94.3.236 18943971

[B30] Cao Z. Li L. Kapoor K. Banniza S. (2019). Using a transcriptome sequencing approach to explore candidate resistance genes against stemphylium blight in the wild lentil species *Lens ervoides* . BMC Plant Biol. 19, 399. doi: 10.1186/s12870-019-2013-6 31510924 PMC6740027

[B31] Caprioli G. Cristalli G. Ragazzi E. Molin L. Ricciutelli M. Sagratini G.. (2010). A preliminary matrix-assisted laser desorption/ionization time-of-flight approach for the characterization of Italian lentil varieties. Rapid Commun. Mass Spectrom. 24, 2843–2848. doi: 10.1002/rcm.4711 20857444

[B32] Chen Y. H. Shapiro L. R. Benrey B. Cibrián-Jaramillo A. (2017). Back to the origin: *In situ* studies are needed to understand selection during crop diversification. Front. Ecol. Evol. 5, 1–8. doi: 10.3389/fevo.2017.00125

[B33] Cheung F. Haas B. J. Goldberg S. M. D. May G. D. Xiao Y. Town C. D. (2006). Sequencing *Medicago truncatula* expressed sequenced tags using 454 life sciences technology. BMC Genom. 7, 272. doi: 10.1186/1471-2164-7-272 PMC1635983 17062153

[B34] Civantos-Gómez I. Rubio Teso M. L. Galeano J. Rubiales D. Iriondo J. M. Garc´ıa-Algarra J. (2022). Climate change conditions the selection of rust-resistant candidate wild lentil populations for *in situ* conservation. Front. Plant Sci. 13. doi: 10.3389/fpls.2022.1010799 PMC9669080 36407589

[B1200] Climate.gov. (2022). Climate Change 2022: Impacts, Adaptation and Vulnerability. Available at: https://www.ipcc.ch/2022/02/28/pr-wgii-ar6/.

[B35] Cortinovis G. Oppermann M. Neumann K. Graner A. Gioia T. Marsella M.. (2021). Towards the development, maintenance, and standardized phenotypic characterization of single-seed-descent genetic resources for common bean. Curr. Protoc. 1, e133. doi: 10.1002/cpz1.133 34004060

[B36] Coyne C. J. Kumar S. von Wettberg E. J. Marques E. Berger J. D. Redden R. J.. (2020). Potential and limits of exploitation of crop wild relatives for pea, lentil, and chickpea improvement. Legume Sci. 2, e36. doi: 10.1002/leg3.36

[B37] Cristobal M. D. Pando V. Herrero B. (2014). Morphological characterization of lentil (*Lens culinaris* medik.) landraces from castilla y león, Spain. Pak. J. Bot. 46, 1373–1380.

[B38] Cubero J. I. (1981). “Origin, taxonomy and domestication,” in Lentils. Eds. Webb C. Hawtin G. (London: Commonwealth Agricultural Bureaux), 15–38.

[B39] Dadu R. H. R. Bar I. Ford R. Sambasivam P. Croser J. Ribalta F.. (2021). *Lens orientalis* contributes quantitative trait loci and candidate genes associated with ascochyta blight resistance in lentil. Front. Plant Sci. 12. doi: 10.3389/fpls.2021.703283 PMC8442733 34539696

[B1001] Dash S. Campbell J. D. Cannon E. K. Cleary A. M. Huang W. Kalberer S. R.. (2016). Legume information system (LegumeInfo.org): A key component of a set of federated data resources for the legume family. Nucleic Acids Research 44(D1), D1181–D1188. doi: 10.1093/nar/gkv1159 26546515 PMC4702835

[B40] de la Puente R. Garcia P. Polanco C. Perez de la Vega M. (2013). An improved intersubspecific genetic map in *Lens* including functional markers. Span. J. Agric. Res. 11, 132–136. doi: 10.5424/sjar/2013111-3283

[B41] Dhakate P. Sehgal D. Vaishnavi S. Chandra A. Singh A. Raina S. N.. (2022). Comprehending the evolution of gene editing platforms for crop trait improvement. Front. Genet. 13. doi: 10.3389/fgene.2022.876987 PMC9445674 36082000

[B42] Dissanayake R. Kahrood H. V. Dimech A. M. Noy D. M. Rosewarne G. M. Smith K. F.. (2020). Development and application of image-based high-throughput phenotyping methodology for salt tolerance in lentils. Agronomy 10, 1992–2008. doi: 10.3390/agronomy10121992

[B43] Dixit G. P. Katiyar P. K. Singh B. B. Kumar S. (2009). Lentil varieties in india. all India coordinated research project on MULLaRP (Kanpur, India: IIPR), p13.

[B44] Doyle J. J. (1988). 5S ribosomal gene variation in the soybean and its progenitor. Theor. Appl. Genet. 75, 621–624. doi: 10.1007/BF00289130

[B45] Dubos N. Montfort F. Grinand C. Nourtier M. Deso G. Probst J. M.. (2022). Are narrow-ranging species doomed to extinction? projected dramatic decline in future climate suitability of two highly threatened species. Perspect. Ecol. Conserv. 20, 18–28. doi: 10.1016/j.pecon.2021.10.002

[B46] Duke J. A. (1981). Handbook of legumes of world economic importance (New York: Plenum Press).

[B47] Durán Y. Fratini R. Garcia P. de la Vega M. P. (2004). An intersubspecific genetic map of *Lens* .Theor. Appl. Genet. 108, 1265–1273. doi: 10.1007/s00122-003-1542-3 14676948

[B48] El-Bouhssini M. Sarker A. Erskine W. Joubi A. (2008). First sources of resistance to *Sitona* weevil (*Sitona crinitus* herbst.) in wild *Lens* species. Genet. Resour. Crop Evol. 55, 1–4. doi: 10.1007/s10722-007-9297-3

[B49] Erskine W. Chandra S. Chaudhry M.. (1998). A bottleneck in lentil: widening its genetic base in south Asia. Euphytica 101, 207–211. doi: 10.1023/A:1018306723777

[B50] Eujayl I. Baum M. Powell W. Erskine W. Pehu E. (1998). A genetic linkage map of lentil (*Lens* sp.) based on RAPD and AFLP markers using recombinant inbred lines. Theor. Appl. Genet. 97, 83–89. doi: 10.1007/s001220050869

[B51] Evolves (2019). Available at: https://knowpulse.usask.ca/study/2691111 (Accessed September 15, 2022).

[B52] Fang Y. Xiong L. (2015). General mechanisms of drought response and their application in drought resistance improvement in plants. Cell. Mol. Life Sci. 72, 673–689. doi: 10.1007/s00018-014-1767-0 25336153 PMC11113132

[B53] FAOSTAT (2019). Available at: https://www.fao.org/2019 (Accessed September 15, 2022).

[B54] FAOSTAT (2020). Available at: https://www.fao.org/2019 (Accessed September 15, 2022).

[B55] Fedoruk M. J. Vandenberg A. Bett K. E. (2013). Quantitative trait loci analysis of seed quality characteristics in lentil using single nucleotide polymorphism markers. Plant Genome 6, plantgenome2013.05.0012. doi: 10.3835/plantgenome2013.05.0012 PMC12962556 29505642

[B56] Ferguson M. E. Erskine W. (2001). “Lentiles (L.),” in Plant genetic resources of legumes in the Mediterranean. Eds. Maxted N. Bennett S. J. (Dordrecht, The Netherlands: Kluwer Academic Publishers), 132–157.

[B57] Ferguson M. E. Maxted N. Van Slageren M. Robertson L. D. (2000). A re-assessment of the taxonomy of *Lens* mill. (Leguminosae, papilionoideae, vicieae). Bot. J. Linn. Soc 133, 41–59. doi: 10.1111/j.1095-8339.2000.tb01536.x

[B58] Ferguson M. E. Robertson L. D. Ford-Lloyd B. V. Newbury H. J. Maxted N. (1998). Contrasting genetic variation amongst lentil landraces from different geographical origins. Euphytica 102, 265–273. doi: 10.1023/A:1018331432580

[B59] Fernández-Aparicio M. Sillero J. C. Rubiales D. (2009). Resistance to broomrape in wild lentils (*Lens* spp.). Plant Breed. 128, 266–270. doi: 10.1111/j.1439-0523.2008.01559.x

[B60] Fiala J. V. Tullu A. Banniza S. Séguin-Swartz G. Vandenberg A. (2009). Interspecies transfer of resistance to anthracnose in lentil (*Lens culinaris* medic.). Crop Sci. 49, 825–830. doi: 10.2135/cropsci2008.05.0260

[B61] Fikiru E. Tesfaye K. Bekele E. (2007). Genetic diversity and population structure of Ethiopian lentil (*Lens culinaris* medikus) landraces as revealed by ISSR marker. Afr. J. Biotechnol. 6, 1460–1468.

[B67] Ford R. Pang E. Taylor P. (1997). Diversity analysis and species identification in *Lens* using PCR generated markers. Euphytica 96, 247–255. doi: 10.1023/A:1003097600701

[B68] Ford R. Taylor P. W. J. (2003). Construction of an intraspecific linkage map of lentil (*Lens culinaris* ssp. *culinaris*). Theor. Appl. Genet. 107, 910–916. doi: 10.1007/s00122-003-1326-9 12830386

[B62] Fratini R. Durán Y. García P. Pérez de la Vega M. (2007). Identification of quantitative trait loci (QTL) for plant structure, growth habit and yield in lentil. Span. J. Agric. Res. 5, 348–356. doi: 10.5424/sjar/2007053-255

[B63] Fratini R. Garcia P. Ruiz M. L. (2006). Pollen and pistil morphology, *in vitro* pollen grain germination and crossing success of *Lens* cultivars and species. Plant Breed. 125, 501–505. doi: 10.1111/j.1439-0523.2006.01277.x

[B64] Fratini R. Ruiz M. L. (2006). Interspecific hybridization in the *Lens* applying *in vitro* embryo rescue. Euphytica 150, 271–280. doi: 10.1007/s10681-006-9118-3

[B65] Fratini R. Ruiz M. L. (2011) “Wide crossing in lentil through embryo rescue,” in Plant embryo culture Eds. Thorpe T. A. Young E. C. (New York: Humana press), 131–139. 10.1007/978-1-61737-988-8_11 21207267

[B66] Fratini R. Ruiz M. L. de la Vega M. P. (2004). Intra-specific and inter-sub-specific crossing in lentil (*Lens culinaris* medik.). Can. J. Plant Sci. 84, 981–986. doi: 10.4141/P03-20

[B69] Galushko V. Gamtessa S. (2022). Impact of climate change on productivity and technical efficiency in Canadian crop production. Sustainability 14, 4241–4262. doi: 10.3390/su14074241

[B71] García-García I. Méndez-Cea B. Martín-Gálvez D. Seco J. I. Gallego F. J. (2021). And linares, J Challenges and perspectives in the epigenetics of climate change-induced forests decline. C.Front. Plant Sci. 12. doi: 10.3389/fpls.2021.797958 PMC8764141 35058957

[B70] García-García P. Vaquero F. Vences F. J. Sáenz de Miera L. E. Polanco C. González A. I.. (2019). Transcriptome profiling of lentil in response to *Ascochyta lentis* infection. Span. J. Agric. Res. 17, 14982. doi: 10.5424/sjar/2019174-14982

[B72] Gela T. S. Koh C. S. Caron C. T. Chen L. A. Vandenberg A. Bett K. E. (2021a). QTL mapping of lentil anthracnose (*Colletotrichum lentis*) resistance from lens ervoides accession IG 72815 in an interspecific RIL population. Euphytica 217, 1–11. doi: 10.1007/s10681-021-02804-0

[B73] Gela T. Ramsay L. Haile T. A. Vandenberg A. Bett K. (2021b). Identification of anthracnose race 1 resistance loci in lentil by integrating linkage mapping and genome-wide association study. Plant Genome 14, e20131. doi: 10.1002/tpg2.20131 34482633 PMC12806994

[B74] Gordeev R. V. Pyzhev A. I. Zander E. V. (2022). Does climate change influence Russian agriculture? evidence from panel data analysis. Sustainability 14, 718. doi: 10.3390/su14020718

[B75] Gorim L. Y. Vandenberg A. (2017). Evaluation of wild lentil species as genetic resources to improve drought tolerance in cultivated lentil. Front. Plant Sci. 8. doi: 10.3389/fpls.2017.01129 PMC5489631 28706524

[B76] Gramazio P. Prohens J. Toppino L. Plazas M. (2021). Editorial: Introgression breeding in cultivated plants. Front. Plant Sci. 12. doi: 10.3389/fpls.2021.764533 PMC8505732 34650586

[B77] Guerra-García A. Gioia T. von Wettberg E. Logozzo G. Papa R. Bitocchi E.. (2021). Intelligent characterization of lentil genetic resources: Evolutionary history, genetic diversity of germplasm, and the need for well-represented collections. Curr. Protoc. 1, e134. doi: 10.1002/cpz1.134 34004055

[B78] Gujaria-verma N. Vail S. L. Carrasquilla-garcia N. Penmetsa R. Cook D. R. Farmer A. D.. (2014). Genetic mapping of legume orthologs reveals high conservation of synteny between lentil species and the sequenced genomes of *Medicago* and chickpea. Front. Plant Sci. 5. doi: 10.3389/fpls.2014.00676 PMC4256995 25538716

[B81] Gupta R. Mishra A. (2019). Climate change induced impact and uncertainty of rice yield of agro-ecological zones of India. Agric. Syst. 173, 1–11. doi: 10.1016/j.agsy.2019.01.009

[B83] Gupta D. Sharma S. K. (2005). Embryo-ovule rescue technique overcoming post-fertilization barriers in inter-specific crosses of *Lens* . J. lentil Res. 2, 27–30.

[B80] Gupta D. Sharma S. K. (2006). Evaluation of wild *Lens* taxa for agromorphological traits, fungal diseases and moisture stress in northwestern Indian hills. Genet. Resour. Crop Evol. 53, 1233–1241. doi: 10.1007/s10722-005-2932-y

[B84] Gupta D. Sharma S. K. (2007). Widening the gene pool of cultivated lentils through introgression of alien chromatin from wild *Lens* subspecies. Plant Breed. 126, 58–61. doi: 10.1111/j.1439-0523.2007.01318.x

[B82] Gupta S. Singh B. B. (2009). “Utilization of potential germplasm and species spectrum in improvement of pulse crops,” in Legumes for ecological sustainability. Eds. Ali M. Gupta S. Basu P. S. Naimuddin K. (Kanpur, India: Indian Institute of Pulse Research), 332–341.

[B85] Gupta D. Taylor P. W. J. Inder P. Phan H. T. T. Ellwood S. R. Mathur P. N.. (2012a). Integration of EST-SSR markers of medicago trunculata into intraspecific linkage map of lentil and identification of QTL conferring resistance to ascochyta blight at seedling and pod stages. Mol. Breed. 30, 429–439. doi: 10.1007/s11032-011-9634-2

[B86] Gupta M. Verma B. Kumar N. Chahota R. K. Rathour R. Sharma S. K. (2012b). Construction of intersubspecific molecular genetic map of lentil based on ISSR, RAPD and SSR markers. J. Genet. 91, 279–287. doi: 10.1007/s12041-012-0180-4 23271013

[B79] Gutierrez-Gonzalez J. J. García P. Polanco C. González A. I. Vaquero F. Vences F. J.. (2022). Multi-species transcriptome assemblies of cultivated and wild lentils (*Lens* sp.) provide a first glimpse at the lentil pangenome. Agronomy 12, 1619–27. doi: 10.3390/agronomy12071619

[B88] Hamdi A. Erskine W. (1996). Reaction of wild species of the genus *Lens* to drought. Euphytica 91, 173–179. doi: 10.1007/BF00021067

[B89] Hamdi A. Küsmenoĝlu I. Erskine W. (1996). Sources of winter hardiness in wild lentil. Genet. Resour. Crop Evol. 43, 63–67. doi: 10.1007/BF00126942

[B90] Hamwieh A. Udupa S. M. Choumane W. Sarker A. Dreyer F. Jung C.. (2005). A genetic linkage map of lens sp. based on microsatellite and AFLP markers and the localization of fusarium vascular wilt resistance. Theor. Appl. Genet. 110, 669–677. doi: 10.1007/s00122-004-1892-5 15650814

[B91] Hamwieh A. Udupa S. M. Sarker A. Jung C. Baum M. (2009). Development of new microsatellite markers and their application in the analysis of genetic diversity in lentils. Breed. Sci. 59, 77–86. doi: 10.1270/jsbbs.59.77

[B92] Hansen J. Renfrew J. M. (1978). Palaeolithic–Neolithic seed remains at franchthi cave, Greece. Nature 271, 349–352. doi: 10.1038/271349a0

[B93] Hao M. Zhang L. Ning S. Huang L. Yuan Z. Wu B.. (2020). The resurgence of introgression breeding, as exemplified in wheat improvement. Front. Plant Sci. 11. doi: 10.3389/fpls.2020.00252 PMC7067975 32211007

[B94] Harlan J. R. (1992). Crops and man’ (Madison, Wisconsin, USA: American Society of Agronomy).

[B87] Havey M. J. Muehlbauer F. J. (1989). Variability for restriction fragment lengths and phylogenies in lentil. Theor. Appl. Genet. 77, 839–843. doi: 10.1007/BF00268336 24232901

[B95] Heineck G. C. Altendorf K. R. Coyne C. J. Ma Y. McGee R. Porter L. D. (2022). Phenotypic and genetic characterization of the lentil single plant-derived core collection for resistance to root rot caused by *Fusarium avenaceum* . Phytopathology 112, 1979–1987. doi: 10.1094/PHYTO-12-21-0517-R 35657701

[B96] Huang X. Huang S. Han B. Li J. (2022). The integrated genomics of crop domestication and breeding. Cell 185 (15), 2828–2839. doi: 10.1016/j.cell.2022.04.036 35643084

[B97] Hussain S. A. Iqbal M. S. Akbar M. Arshad N. Munir S. Ali M. A.. (2022). Estimating genetic variability among diverse lentil collections through novel multivariate techniques. PloS One 17, e0269177. doi: 10.1371/journal.pone.0269177 35771871 PMC9246128

[B99] Idrissi O. Udupa S. M. De Keyser E. McGee R. J. Coyne C. J. Saha G. C.. (2016). Identification of quantitative trait loci controlling root and shoot traits associated with drought tolerance in a lentil (*Lens culinaris* medik.) recombinant inbred line population. Front. Plant Sci. 7. doi: 10.3389/fpls.2016.01174 PMC4993778 27602034

[B98] Idrissi O. Udupa S. M. Houasli C. De Keyser E. Van Damme P. De Riek J. (2015). Genetic diversity analysis of Moroccan lentil (*Lens culinaris* medik.) landraces using simple sequence repeat and amplified fragment length polymorphisms reveals functional adaptation towards agro-environmental origins. Plant Breed. 134, 322–332. doi: 10.1111/pbr.12261

[B100] Iizumi T. Shiogama H. Imada Y. Hanasaki N. Takikawa H. Nishimori M. (2018). Crop production losses associated with anthropogenic climate change for 1981-2010 compared with preindustrial levels. Int. J. Climatol 38, 5405–5417. doi: 10.1002/joc.5818

[B101] INCREASE. Available at: https://www.pulsesincrease.eu/crops/lentil (Accessed September 15, 2022).

[B102] IPCC. Available at: www.climate.gov/2022 (Accessed September 15, 2022).

[B103] Janzen G. M. Wang L. Hufford M. B. (2019). The extent of adaptive wild introgression in crops. New Phytol. 22, 1279–1288. doi: 10.1111/nph.15457 30368812

[B104] Jarvis A. Lane A. Hijmans R. J. (2008). The effect of climate change on crop wild relatives. Agric. Ecosyst. Environ. 126, 13–23. doi: 10.1016/j.agee.2008.01.013

[B105] Johnson N. Boatwright J. L. Bridges W. Thavarajah P. Kumar S. Shipe E.. (2021). Genome-wide association mapping of lentil (*Lens culinaris* medikus) prebiotic carbohydrates toward improved human health and crop stress tolerance. Sci. Rep. 11, 1–12. doi: 10.1038/s41598-021-93475-3 34230595 PMC8260633

[B106] Juroszek P. Racca P. Link S. Farhumand J. Kleinhenz B. (2020). Overview on the review articles published during the past 30 years relating to the potential climate change effects on plant pathogens and crop disease risks. Plant Pathol. 69, 179–193. doi: 10.1111/ppa.13119

[B107] Kadiyala M. D. Nedumaran S. Padmanabhan J. Gumma M. K. Gummadi S. Srigiri S. R.. (2021). Modeling the potential impacts of climate change and adaptation strategies on groundnut production in India. Sci. Total Environ. 776, 145996. doi: 10.1016/j.scitotenv.2021.145996

[B110] Kahraman A. Pandey A. Khan M. K. Lindsay D. Moenga S. Vance L.. (2017). Distinct subgroups of cicer echinospermum are associated with hybrid sterility and breakdown in interspecific crosses with cultivated chickpea. Crop Sci. 57 (6), pp.3101–3111. doi: 10.2135/cropsci2017.06.0335

[B108] Kahraman A. Demirel U. Ozden M. Muehlbauer F. J. (2010). Mapping of QTLs for leaf area and the association with winter hardiness in fall-sown lentil. Afr. J. Biotechnol. 9, 8515–8519. doi: 10.5897/AJB10.57

[B109] Kahraman A. Kusmenoglu I. Aydin N. Aydogan A. Erskine W. Muehlbauer F. J. (2004). QTL mapping of winter hardiness genes in lentil. Crop Sci. 44, 13–22. doi: 10.2135/cropsci2004.1300

[B111] Kahraman A. Temel H. Y. Aydogan A. Tanyolac M. B. (2015). Major quantitative trait loci for flowering time in lentil. Turk J. Agric. For 39, 588–595. doi: 10.3906/tar-1408-16

[B112] Kant C. Pandey V. Verma S. Tiwari M. Kumar S. Bhatia S. (2017). “Transcriptome analysis in chickpea (Cicer arietinum l.): Applications in study of gene expression, non-coding RNA prediction, and molecular marker development,” in Applications of RNA-seq and omics strategies - from microorganisms to human health. Eds. Marchi FA F. A. PDR C. Mateo E. C. (IntechOpen), 245–263.

[B113] Kaur S. Cogan N. O. I. Pembleton L. W. Shinozuka M. Savin K. W. Materne M.. (2011). Transcriptome sequencing of lentil based on second-generation technology permits large-scale unigene assembly and SSR marker discovery. BMC Genom. 12, 265. doi: 10.1186/1471-2164-12-265 PMC3113791 21609489

[B114] Kaur S. Cogan N. O. Stephens A. Noy D. Butsch M. Forster J. W.. (2014). EST-SNP discovery and dense genetic mapping in lentil (*Lens culinaris* medik.) enable candidate gene selection for boron tolerance. Theor. Appl. Genet. 127, 703–713. doi: 10.1007/s00122-013-2252-0 24370962

[B115] Khan M. K. Pandey A. Athar T. Hamurcu M. Gezgin S. Sassi G.. (2022). “Current trends in genetic enhancement of legumes in the genomics era for a sustainable future,” in Advances in legumes for sustainable intensification (Academic Press), (pp. 533–552).

[B116] Khazaei H. Caron C. T. Fedoruk M. Diapari M. Vandenberg A. Coyne C. J.. (2016). Genetic diversity of cultivated lentil (*Lens culinaris* medik.) and its relation to the world’s agro-ecological zones. Front. Plant Sci. 7. doi: 10.3389/fpls.2016.01093 PMC4960256 27507980

[B118] Khazaei H. Fedoruk M. Caron C. T. Vandenberg A. Bett K. E. (2018). Single nucleotide polymorphism markers associated with seed quality characteristics of cultivated lentil. Plant Genome 11, 170051. doi: 10.3835/plantgenome2017.06.0051 PMC12962556 29505642

[B117] Khazaei H. Podder R. Caron C. T. Kundu S. S. Diapari M. Vandenberg A.. (2017). Marker–trait association analysis of iron and zinc concentration in lentil (*Lens culinaris* medik.) seeds. Plant Genome 10, plantgenome2017.02.0007. doi: 10.3835/plantgenome2017.02.0007 28724070

[B119] Khorramdelazad M. Bar I. Whatmore P. Smetham G. Bhaaskaria V. Yang Y.. (2019). Transcriptome profiling of lentil (Lens culinaris) through the first 24 hours of ascochyta lentis infection reveals key defence response genes. BMC Genomics 19, 108. doi: 10.1186/s12864-018-4488-1 PMC5793396 29385986

[B121] Koul P. M. Sharma V. Rana M. Chahota R. K. Kumar S. Sharma T. R. (2017). Analysis of genetic structure and interrelationships in lentil species using morphological and SSR markers. 3 Biotech. 7, 83. doi: 10.1007/s13205-017-0683-z PMC5429312 28500404

[B120] Kroc M. Tomazewska M. Czepiel K. Bitocchi E. Oppermann M. Newmann K.. (2021). Lupin INCREASE intelligent collections: Characterization and development of single seed descent genetic resources. Curr. Opin. Plant Biol. 1(7), e191. doi: 10.1002/cpz1.191"10.1002/cpz1.191 34242495

[B123] Kumar S. Barpete S. Kumar J. Gupta P. Sarker A. (2013). Global lentil production: constraints and strategies. SATSA Mukhapatra Annual Tech. Issue 17, 1–13.

[B124] Kumar S. Choudhary A. K. Rana K. S. Sarker A. Singh M. (2018). Biofortification potential of global wild annual lentil core collection. PloS One 13, e0191122. doi: 10.1371/journal.pone.0191122 29346404 PMC5773171

[B122] Kumar J. Gupta D. S. Baum M. Varshney R. K. Kumar S. (2021). Genomics-assisted lentil breeding: current status and future strategies. Legume Sci. 3, e71. doi: 10.1002/leg3.71

[B125] Kumar S. Rajendran K. Kumar J. Hamwieh A. Baum M. (2015). Current knowledge in lentil genomics and its application for crop improvement. Front. Plant Sci. 6. doi: 10.3389/fpls.2015.00078 PMC4337236 25755659

[B128] Kumar R. Sharma S. K. Sharma A. Sharma S. (2004). Path coefficient analysis of seed yield components in lentil (*Lens culinaris* medik.). Legume Res. 27, 305–307.

[B126] Kumar H. Singh A. Dikshit H. K. Mishra G. P. Aski M. Meena M. C.. (2019). Genetic dissection of grain iron and zinc concentrations in lentil (*Lens culinaris* medik.). J. Genet. 98, 66. doi: 10.1007/s12041-019-1112-3 31544775

[B127] Kumar J. Srivastava E. Singh M. Kumar S. Nadarajan N. Sarker A. (2014). Diversification of indigenous gene-pool by using exotic germplasm in lentil (*Lens culinaris* medikus ssp. *culinaris*). Physiol. Mol. Biol. Plants 20, 125–132. doi: 10.1007/s12298-013-0214-2 24554846 PMC3925481

[B129] Kushwaha U. K. S. Ghimire S. K. Yadav N. K. Ojha B. R. Niroula R. K. (2015). Genetic characterization of lentil (*Lens culinaris* l.) germplasm by using SSR markers. Agri Biol. Sci. J. 1, 16–26.

[B130] Ladizinsky G. (1979). The origin of lentil and its wild gene pool. Euphytica 28, 179–187. doi: 10.1007/BF00029189

[B131] Ladizinsky G. (1993). Wild lentils. Crit. Rev. Plant Sci. 12, 169–184. doi: 10.1080/07352689309701900

[B132] Ladizinsky G. (1997). A new species of *Lens* from south-east Turkey. Bot. J. Linn. Soc. 123, 257–260. doi: 10.1006/bojl.1996.0081

[B133] Ladizinsky G. (1999). Identification of the lentil’s wild genetic stock. Genet. Resour. Crop Evol. 46, 115–118. doi: 10.1023/A:1008626128871

[B134] Ladizinsky G. Braun D. Goshen D. Muehlbauer F. J. (1984). The biological species of the *Lens* l. Bot. Gaz. 145, 253–261. doi: 10.1086/337454

[B135] Laserna-Ruiz I. De-Los-Mozos-Pascual M. Santana-Méridas O. Sánchez-Vioque R. Rodríguez-Conde M. F. (2012). Screening and selection of lentil (*Lens* miller) germplasm resistant to seed bruchids (*Bruchus* spp.). Euphytica 188, 153–162. doi: 10.1007/s10681-012-0752-7

[B136] Leisner C. P. (2020). Review: Climate change impacts on food security- focus on perennial cropping systems and nutritional value. Plant Sci. 293, 110412. doi: 10.1016/j.plantsci.2020.110412 32081261

[B137] Liber M. Oliveira H. R. Duarte I. Maia A. T. (2021). The history of lentil (*Lens culinaris* ssp. *culinaris*) domestication and spread as revealed by genotyping-by-sequencing of wild and landrace accessions. Front. Plant Sci. 12. doi: 10.3389/fpls.2021.628439 PMC8030269 33841458

[B138] Lychuk T. E. Moulin A. P. Lemke R. L. Izaurralde R. C. Johnson E. N. Olfert O. O.. (2021). Modelling the effects of climate change, agricultural inputs, cropping diversity, and environment on soil nitrogen and phosphorus: A case study in Saskatchewan, Canada. Agric. Water Manage. 252, 106850. doi: 10.1016/j.agwat.2021.106850

[B139] Ma Y. Marzougui A. Coyne C. J. Sankaran S. Main D. Porter L. D.. (2020). Dissecting the genetic architecture of aphanomyces root rot resistance in lentil by QTL mapping and genome-wide association study. Int. J. Mol. Sci. 21, 2129–2153. doi: 10.3390/ijms21062129 32244875 PMC7139309

[B140] Maghuly F. Molin E. M. Saxena R. Konkin D. J. (2022). Functional genomics in plant breeding 2.0. Int. J. Mol. Sci. 23, 6959–63. doi: 10.3390/ijms23136959 35805968 PMC9266731

[B141] Malhotra N. Panatu S. Singh B. Negi N. Singh D. Singh M.. (2019). “Genetic resources: collection, conservation, characterization and maintenance,” in Lentils: potential resources for enhancing genetic gains. Ed. Singh M.(London: Academic Press), 21–41.

[B142] Mane R. Katoch M. Singh M. Sharma R. Sharma T. R. Chahota R. K. (2020). Identification of genomic regions associated with early plant vigour in lentil (*Lens culinaris*). J. Genet. 99, 1–8. doi: 10.1007/s12041-020-1182-2 32366732

[B143] Materne M. McNeil D. L. (2007). “Breeding methods and achievements,” in Lentil: an ancient crop for modern times. Eds. Yadav S. S. McNeil D. Stevenson P. C. (Dordrecht: Springer), 241–253.

[B144] Mayer M. S. Soltis P. S. (1994). Chloroplast DNA phylogeny of *Lens* (Leguminosae): origin and diversity of the cultivated lentil. Theor. Appl. Genet. 87, 773–781. doi: 10.1007/BF00221128 24190462

[B145] Mekonnen F. Mekbib F. Kumar S. Ahmed S. Sharma T. R. (2015). Correlation and path coefficient analysis of seed yield and yield components in lentil (*Lens culinaris* medik.) genotype in ethiopia. Afr. J. Plant Sci. 8, 507–520. doi: 10.5897/AJPS2014.1183

[B146] Mir R. R. Reynolds M. Pinto F. Khan M. A. Bhat M. A. (2019). High-throughput phenotyping for crop improvement in the genomics era. Plant Sci. 282, 60–72. doi: 10.1016/j.plantsci.2019.01.007 31003612

[B147] Mishra D. Shekhar S. Chakraborty S. Chakraborty N. (2021). High temperature stress responses and wheat: impacts and alleviation strategies. Environ. Exp. Bot. 190, 104589. doi: 10.1016/j.envexpbot.2021.104589

[B148] Morgil H. Tardu M. Cevahir G. Kavakli İ.H. (2019). Comparative RNA-seq analysis of the drought-sensitive lentil (*Lens culinaris*) root and leaf under short- and long-term water deficits. Funct. Integr. Genomics 19, 715–727. doi: 10.1007/s10142-019-00675-2 31001704

[B149] Mourad A. M. Belamkar V. Baenziger P. S. (2020). Molecular genetic analysis of spring wheat core collection using genetic diversity, population structure, and linkage disequilibrium. BMC Genom. 21, 1–2. doi: 10.1186/s12864-020-06835-0 PMC7318758 32586286

[B151] Muehlbauer F. J. Cho S. Sarker A. McPhee K. E. Coyne C. J. Rajesh P. N.. (2006). Application of biotechnology in breeding lentil for resistance to biotic and abiotic stress. Euphytica 147, 149–165. doi: 10.1007/s10681-006-7108-0

[B150] Muehlbauer F. J. McPhee K. E. (2005). “Lentil (L. culinaris medik.),” in Genetic resources and chromosome engineering and crop improvement. grain legumes. Eds. Ram J. S. Prem P. J. (CRC Press Florida), 219–230.

[B152] Muench D. G. Slinkard A. E. Scoles G. J. (1991). Determination of genetic variation and taxonomy in lentil (*Lens* miller) species by chloroplast DNA polymorphism. Euphytica 56, 213–218. doi: 10.1007/BF00042366

[B153] Muscolo A. Junker A. Klukas C. Weigelt-Fischer K. Riewe D. Altmann T. (2015). Phenotypic and metabolic responses to drought and salinity of four contrasting lentil accessions. J. Expt. Bot. 66, 5467–5480. doi: 10.1093/jxb/erv208 PMC4585415 25969553

[B154] Neupane S. (2019). Flowering time response of diverse lentil (Lens culinaris medik.) germplasm grown in multiple environments (Doctoral dissertation, University of Saskatchewan).

[B155] Nguyen C. T. Scrimgeour F. (2022). Measuring the impact of climate change on agriculture in Vietnam: A panel ricardian analysis. Agric. Econ. 53, 37–51. doi: 10.1111/agec.12677

[B156] Ntiamoah E. B. Li D. Appiah-Otoo I. Twumasi M. A. Yeboah E. N. (2022). Towards a sustainable food production: modelling the impacts of climate change on maize and soybean production in Ghana. Environ. Sci. pollut. Res. 29, 72777–72796. doi: 10.1007/s11356-022-20962-z PMC9130696 35610457

[B158] Ogutcen E. Ramsay L. von Wettberg E. B. Bett K. E. (2018). Capturing variation in *Lens* (Fabaceae): Development and utility of an exome capture array for lentil. Appl. Plant Sci. 6, e01165. doi: 10.1002/aps3.1165 30131907 PMC6055568

[B159] Omar G. I. Saqer M. M. Adwan G. M. (2019). Phylogenetic relationship among some species of the genera *Lens*, *Vicia*, *Lathyrus* and *Pisum* (Leguminosae) in Palestine. Jordan J. Biol. Sci. 12, 296.

[B160] Pavan S. Bardaro N. Fanelli V. Marcotrigiano A. R. Mangini G. Taranto. (2019). Genotyping by sequencing of cultivated lentil (Lens culinaris medik.) highlights population structure in the Mediterranean gene pool associated with geographic patterns and phenotypic variables. Front. Genet. 10. doi: 10.3389/fgene.2019.00872 PMC6759463 31620173

[B162] Phan H. T. Ellwood S. R. Hane J. K. Ford R. Materne M. Oliver R. P. (2007). Extensive macrosynteny between *Medicago truncatula* and *Lens culinaris* ssp. *culinaris* . Theor. Appl. Genet. 114, 549–558. doi: 10.1007/s00122-006-0455-3 17119911

[B163] Pielke R. Burgess M. G. Ritchie J. (2022). Plausible 2005–2050 emissions scenarios project between 2° c and 3° c of warming by 2100. Environ. Res. Lett. 17, 024027. doi: 10.1088/1748-9326/ac4ebf

[B164] Podder R. Banniza S. Vandenberg A. (2013). Screening of wild and cultivated lentil germplasm for resistance to stemphylium blight. Plant Genet. Resour. 11, 26–35. doi: 10.1017/S1479262112000329

[B165] Polanco C. Sáenz de Miera L. E. González A. I. García P. Fratini R. Vaquero F.. (2019). Construction of a high-density interspecific (*Lens culinaris* x *L. odemensis*) genetic map based on functional markers for mapping morphological and agronomical traits, and QTLs affecting resistance to ascochyta in lentil. PloS One 14, e0214409. doi: 10.1371/journal.pone.0214409 30917174 PMC6436743

[B167] Pratap A. Das A. Kumar S. Gupta S. (2021). Current perspectives on introgression breeding in food legumes. Front. Plant Sci. 11. doi: 10.3389/fpls.2020.589189 PMC7858677 33552095

[B166] Pratap A. Gupta S. K. (2009). “Biotechnological interventions in host plant resistance,” in Integrated pest management: Innovation, dissemination and impac. Eds. Peshin R. Dhawan A. K. (Dordrecht: Springer), 183–207. doi: 10.1007/978-1-4020-8992-3_8

[B157] Ogutcen E. Pandey A. Khan M. K. Khan E. Penmetsa R.V. Kahraman A.. Pod shattering: a homologous series of variation underlying domestication and an avenue for crop improvement. Agron. 8 8, 137. doi: 10.3390/agronomy8080137

[B168] Quezada-Martinez D. Addo Nyarko C. P. Schiessl S. V. Mason A. S. (2021). Using wild relatives and related species to build climate resilience in *Brassica* crops. Theor. Appl. Genet. 134, 1711–1728. doi: 10.1007/s00122-021-03793-3 33730183 PMC8205867

[B169] Rajandran V. Ortega R. Vander Schoor J. K. Butler J. B. Freeman J. S. Hecht V. F. G.. (2022). Genetic analysis of early phenology in lentil identifies distinct loci controlling component traits. J. Exp. Bot. 73, 3963–3977. doi: 10.1093/jxb/erac107 35290451 PMC9238442

[B170] Rajendran K. Coyne C. Zheng P. Saha G. Main D. Amin N.. (2021). Genetic diversity and GWAS of agronomic traits using an ICARDA lentil (Lens culinaris medik.) reference plus collection. Plant Genet. Resources: Characterization Utilization 19 (4), 279–288. doi: 10.1017/S147926212100006X

[B171] Rajpal V. R. Rama Rao S. Raina S. N. (2016a). Gene pool diversity and crop improvement: Vol. 1 (Springer: Cham). doi: 10.1007/978-3-319-27096-8

[B172] Rajpal V. R. RamaRao S. Raina S. N. (2016b). Molecular breeding for sustainable crop improvemen, vol. II (Springer: Cham). doi: 10.1007/978-3-319-27090-6

[B173] Rajpal V. R. Sehgal D. Kumar A. Raina S. N. (2019a). Genetic enhancement of crops for tolerance to abiotic stress: mechanisms and approaches vol. I (Springer: Cham). doi: 10.1007/978-3-319-91956-0

[B174] Rajpal V. R. Sehgal D. Kumar A. Raina S. N. (2019b). Genomics assisted breeding of crops for abiotic stress tolerance vol. II (Springer: Cham). doi: 10.1007/978-3-319-99573-1

[B175] Ramsay L. Chan C. Sharpe A. G. Cook D. R. Penmetsa R. V. Chang P.. (2016) L. culinaris CDC redberry genome assembly v1.2. Available at: https://knowpulse.usask.ca/genome-assembly/Lc1.2.

[B176] Ramsay L. Koh C. S. Kagale S. Gao D. Kaur S. Haile T.. (2021) Genomic rearrangements have consequences for introgression breeding as revealed by genome assemblies of wild and cultivated lentil species. bioRxiv. Available at: https://knowpulse.usask.ca/genome-assembly/Ler.1DRT.

[B177] Raturi D. Chaudhary M. Bhat V. Goel S. Raina S. N. Rajpal V. R.. (2022). Overview of developed core and mini core collections and their effective utilization in cultivated rice and its related species (*Oryza* sp.)-a review. Plant Breed 141, 501–512. doi: 10.1111/pbr.13029

[B178] Reddy M. R. Rathour R. Kumar N. Kathoch P. Sharma T. R. (2009). Crossgenera legume SSR markers for analysis of genetic diversity in *Lens* species. Plant Breed. 129, 514–518. doi: 10.1111/j.1439-0523.2009.01723.x

[B179] Renzi J. P. Coyne C. J. Berger J. Von Wettberg E. Nelson M. Ureta S.. (2022). How could the use of crop wild relatives in breeding increase the adaptation of crops to marginal environments? Front. Plant Sc. 13. doi: 10.3389/fpls.2022.886162 PMC9243378 35783966

[B180] Rodda M. S. Davidson J. Javid M. Sudheesh S. Blake S. Forster J. W.. (2017). Molecular breeding for ascochyta blight resistance in lentil: Current progress and future directions. Front. Plant Sci. 8. doi: 10.3389/fpls.2017.01136 PMC5489742 28706526

[B181] Rodda M. S. Sudheesh S. Javid M. Noy D. Gnanasambandam A. Slater A. T.. (2018). Breeding for boron tolerance in lentil (Lens culinaris medik.) using a high-throughput phenotypic assay and molecular markers. Plant Breed 137, 492–501. doi: 10.1111/pbr.12608

[B182] Román-Palacios C. Wiens J. J. (2020). Recent responses to climate change reveal the drivers of species extinction and survival. Proc. Natl. Acad. Sci. U.S.A. 117, 4211–4217. doi: 10.1073/pnas.1913007117 32041877 PMC7049143

[B183] Roy A. Sahu P. K. Das C. Bhattacharyya S. Raina A. Mondal S. (2023). Conventional and new-breeding technologies for improving disease resistance in lentil (*Lens culinaris* medik). Front. Plant Sci. 13. doi: 10.3389/fpls.2022.1001682 PMC9896981 36743558

[B184] Rubeena F. Taylor P. W. J. (2003). Construction of an intraspecific linkage map of lentil (*Lens culinaris* ssp. culinaris). Theor. Appl. Genet. 107, 910–916. doi: 10.1007/s00122-003-1326-9 12830386

[B185] Rubio Teso M. L. Lara-Romero C. Rubiales D. Parra-Quijano M. Iriondo J. M. (2022). Searching for abiotic tolerant and biotic stress resistant wild lentils for introgression breeding through predictive characterization. Front. Plant Sc. 13. doi: 10.3389/fpls.2022.817849 PMC8928559 35310661

[B186] Saha G. C. Sarker A. Chen W. Vandemark G. J. Muehlbauer F. J. (2010a). Inheritance and linkage map positions of genes conferring resistance to stemphylium blight in lentil. Crop Sci. 50, 1831–1839. doi: 10.2135/cropsci2009.12.0709

[B187] Saha G. C. Sarker A. Chen W. D. Vandemark G. J. Muehlbauer F. J. (2010b). Identification of markers associated with genes for rust resistance in *Lens culinaris* medik. Euphytica 175, 261–265. doi: 10.1007/s10681-010-0187-y

[B188] Saha G. C. Sarker A. Chen W. D. Vandemark G. J. Muehlbauer F. J. (2013). Inheritance and linkage map positions of genes conferring agromorphological traits in *Lens culinaris* medik. Int. J. Agron. 2013, 1–9. doi: 10.1155/2013/618926

[B189] Salaria S. Boatwright J. L. Thavarajah P. Kumar S. Thavarajah D. (2022). Protein biofortification in lentils (*Lens culinaris* medik.) toward human health. Front. Plant Sci. 13. doi: 10.3389/fpls.2022.869713 PMC9016278 35449893

[B190] Salgotra R. K. Stewart C. N. (2022). Genetic augmentation of legume crops using genomic resources and genotyping platforms for nutritional food security. Plants 11, 1866–87. doi: 10.3390/plants11141866 35890499 PMC9325189

[B191] Sanderson L. A. Caron C. T. Tan R. Shen Y. Liu R. Bett K. E. (2019). KnowPulse: a web-resource focused on diversity data for pulse crop improvement. Front. Plant Sci. 10. doi: 10.3389/fpls.2019.00965 PMC6690010 31428111

[B192] Sarker A. Erskine W. (2006). Recent progress in the ancient lentil. J. Agric. Sci. 144, 19–29. doi: 10.1508/cytologia.59.7

[B193] Sarker A. Singh M. Rajaram S. Erskine W. (2010). Adaptation of small seeded red lentil (*Lens culinaris* medik.) to diverse environments. Crop Sci. 50, 1250–1259. doi: 10.2135/cropsci2009.06.0342

[B194] Schaefer H. Hechenleitner P. Santos-Guerra A. de Sequeira M. M. Pennington R. T. Kenicer G.. (2012). Systematics, biogeography, and character evolution of the legume tribe fabeae with special focus on the middle-Atlantic island lineages. BMC Evol. Biol. 12, 1–19. doi: 10.1186/1471-2148-12-250 23267563 PMC3547781

[B196] Scippa G. S. Rocco M. Ialicicco M. Trupiano D. Viscosi V. Di Michele M.. (2010). The proteome of lentil (*Lens culinaris* medik.) seeds: discriminating between landraces. Electrophoresis 31, 497–506. doi: 10.1002/elps.200900459 20119961

[B195] Scippa G. S. Trupiano D. Rocco M. Viscosi V. Di Michele M. D’andrea A.. (2008). An integrated approach to the characterization of two autochthonous lentil (*Lens culinaris*) landraces of molise (south-central Italy). Heredity 101, 136–144. doi: 10.1038/hdy.2008.39 18478027

[B197] Semagn K. Bjornstad A. Skinnes H. Maroy A. G. Tarkegne Y. William M. (2006). Distribution of DArT, AFLP, and SSR markers in a genetic linkage map of a doubled-haploid hexaploid wheat population. Genome 49, 545–555. doi: 10.1139/g06-002 16767179

[B198] Sen Gupta D. Thavarajah D. McGee R. J. Coyne C. J. Kumar S. Thavarajah P. (2016). Genetic diversity among cultivated and wild lentils for iron, zinc, copper, calcium and magnesium concentrations. Aust. J. Crop Sci. 10, 1381–1387. doi: 10.21475/ajcs.2016.10.10.pne6

[B199] Shaheen N. Tahir A. Khan M. A. Ilyas M. K. Ghafoor A. (2022). Estimating genetic variability among diverse lentil collections through novel multivariate techniques. PloS One 17, e0269177. doi: 10.1371/journal.pone.0269177 35771871 PMC9246128

[B200] Shahzad A. Ullah S. Dar A. A. Sardar M. F. Mehmood T. Tufail M. A.. (2021). Nexus on climate change: agriculture and possible solution to cope future climate change stresses. Environ. Sci. pollut. Res. 28, 14211–14232. doi: 10.1007/s11356-021-12649-8 33515149

[B201] Shaikh R. Diederichsen A. Harrington M. Adam J. Conner R. L. Buchwaldt L. (2013). New sources of resistance to *Colletotrichum truncatum* race Ct0 and Ct1 in *Lens culinaris* medikus ssp. *culinaris* obtained by single plant selection in germplasm accessions. Genet. Res. Crop Evol. 60, 193–201. doi: 10.1007/s10722-012-9825-7

[B202] Shao G. Halpin P. N. (1995). Climatic controls of eastern north American coastal tree and shrub distributions. J. Biogeogr. 22, 1083–1089. doi: 10.2307/2845837

[B203] Sharma S. K. Dawson I. K. Waugh R. (1995). Relationships among cultivated and wild lentils revealed by RAPD analysis. Theor. Appl. Genet. 91, 647–654. doi: 10.1007/BF00223292 24169893

[B204] Sharma S. K. Knox M. R. Ellis T. N. (1996). AFLP analysis of the diversity and phylogeny of *Lens* and its comparison with RAPD analysis. Theor. Appl. Genet. 93, 751–758. doi: 10.1007/BF00224072 24162404

[B205] Sharpe A. L. Ramsay L. A. Sanderson M. J. Fedoruk W. E. Clarke R. Li S.. (2013). Ancient orphan crop joins modern era: Gene-based SNP discovery and mapping in lentil. BMC Genom. 14, 192. doi: 10.1186/1471-2164-14-192 PMC3635939 23506258

[B206] Sheehy J. E. Elmido A. Centeno C. Pablico P. (2005). Searching for new plants for climate change. J. Agric. Meteorol. 60, 463–468. doi: 10.2480/agrmet.463

[B207] Sihag S. Punia H. Baloda S. Singal M. Tokas J. (2021). Nano-based fertilizers and pesticides: For precision and sustainable agriculture. J. Nanosci. Nanotechnol. 21, 3351–3366. doi: 10.1166/jnn.2021.19016 34739793

[B221] Singh M. Bisht I. S. Dutta M. Kumar K. Kumar S. Bansal K. C. (2014b). Genetic studies on morpho-phenological traits in lentil (*Lens culinaris* medikus) wide crosses. J. Genet. 93, 561–566. doi: 10.1007/s12041-014-0409-5 25189260

[B220] Singh M. Bisht I. S. Kumar S. Dutta M. Bansal K. C. Karale M.. (2014a). Global wild annual *Lens* collection: A potential resource for lentil genetic base broadening and yield enhancement. PloS One 9, e107781. doi: 10.1371/journal.pone.0107781 25254552 PMC4177869

[B218] Singh J. R. Chung G. H. (2016). Landmark research for pulses improvement. Indian J. Genet. Plant Breed. 76, 399–409. doi: 10.5958/0975-6906.2016.00059.6

[B225] Singh S. P. Debouck D. G. Roca W. W. (1997). Successful interspecific hybridization between phaseouls vulgaris l. and p. costaricensis freytag & debouck. Annu. Rep. Bean Improv. Coop. 40, 40–41.

[B208] Singh A. Ganapathysubramanian B. Singh A. K. Sarkar S. (2016). Machine learning for high-throughput stress phenotyping in plants. Trends Plant Sci. 21, 110–124. doi: 10.1016/j.tplants.2015.10.015 26651918

[B222] Singh M. Kumar S. Basandrai A. K. Basandrai D. Malhotra N. Saxena D. R.. (2020). Evaluation and identification of wild lentil accessions for enhancing genetic gains of cultivated varieties. PloS One 15, e0229554. doi: 10.1371/journal.pone.0229554 32126106 PMC7053756

[B210] Singh B. Malhotra N. Gupta D. (2018). Widening the genetic base of cultivated gene pool following introgression from wild *Lens* taxa. Plant Breed. 137, 447–485. doi: 10.1111/pbr.12615

[B223] Singh M. Rana M. K. Kumar K. Bisht I. S. Dutta M. Gautam N. K.. (2013). Broadening the genetic base of lentil cultivars through inter-sub-specific and interspecific crosses of *Lens* taxa. Plant Breed. 132, 667–675. doi: 10.1111/pbr.12089

[B217] Singh G. Sekhon H. S. Sharma P. (2006). Effect of rhizobium, phosphorus and potash on nodulation and productivity of lentil (*Lens* culinaris medik.). J. Plant Sci. Res. 22, 239–241.

[B224] Singh M. Sharma S. K. Singh B. Malhotra N. Chandora R. Sarker A.. (2018). Widening the genetic base of cultivated gene pool following introgression from wild *Lens* taxa. Plant Breed. 137, 470–485. doi: 10.1111/pbr.12615

[B209] Singh A. K. Singh K. M. Bharati R. C. Chandra N. Bhatt B. P. Pedapati A. (2014). Potential of residual sulfur and zinc nutrition in improving powdery mildew (*Erysiphe trifolii*) disease tolerance of lentil (*Lens culinaris* l.). Commun. Soil Sci. Plant Anal. 45, 2807–2818. doi: 10.1080/00103624.2014.954287

[B211] Singh D. Singh C. K. Taunk J. Gaikwad K. Singh V. Sanwal S. K.. (2022a). Linking genome wide RNA sequencing with physio-biochemical and cytological responses to catalogue key genes and metabolic pathways for alkalinity stress tolerance in lentil (*Lens culinaris* medikus). BMC Plant Biol. 22, 99. doi: 10.1186/s12870-022-03489-w 35247970 PMC8897830

[B212] Singh D. Singh C. K. Taunk J. Jadon V. Pal M. Gaikwad K. (2019). Genome wide transcriptome analysis reveals vital role of heat responsive genes in regulatory mechanisms of lentil (*Lens culinaris* medikus). Sci. Rep. 9, 1–9. doi: 10.1038/s41598-019-49496-0 31506558 PMC6736890

[B214] Singh D. Singh C. K. Taunk J. Tomar R. S. S. Chaturvedi A. K. Gaikwad K.. (2017b). Transcriptome analysis of lentil in response to seedling drought stress. BMC Genom. 18, 206. doi: 10.1186/s12864-017-3596-7 PMC5327544 28241862

[B213] Singh D. Singh C. K. Tomar S. Pal M. (2017a). Genetics and molecular mapping of heat tolerance for seedling survival and pod set in lentil. Crop Sci. 57, 3059–3067. doi: 10.2135/cropsci2017.05.0284

[B215] Singh D. Singh C. K. Tomar R. S. S. Taunk J. Singh R. Maurya S.. (2016). Molecular assortment of *Lens* species with different adaptations to drought conditions using SSR markers. PloS One 11, e0147213. doi: 10.1371/journal.pone.0147213 26808306 PMC4726755

[B219] Singh J. Sirari A. Singh H. Kumar A. Jaidka M. Mandahal K. S.. (2021). Identifying and validating SSR markers linked with rust resistance in lentil (*Lens culinaris*). Plant Breed. 140, 477–485. doi: 10.1111/pbr.12917

[B216] Singh D. Taunk J. Singh C. K. Chaudhary P. Gaikwad K. Yadav R. K.. (2022b). Comparative RNA sequencing for deciphering nodes of multiple abiotic stress tolerance in lentil (*Lens culinaris* medikus). Plant Gene 31, 100373. doi: 10.1016/j.plgene.2022.100373

[B226] Skendžić S. Zovko M. Živković I. P. Lešić V. Lemić D. (2021). The impact of climate change on agricultural insect pests. Insects 12, 440. doi: 10.3390/insects12050440 34066138 PMC8150874

[B227] Skliros D. Kalloniati C. Karalias G. Skaracis G. N. Rennenberg H. Flemetakis E. (2018). Global metabolomics analysis reveals distinctive tolerance mechanisms in different plant organs of lentil (*Lens culinaris*) upon salinity stress. Plant Soil 429, 451–468. doi: 10.1007/s11104-018-3691-9

[B228] Song B. K. Chuah T. S. Tam S. M. Olsen K. M. (2014). Malaysian Weedy rice shows its true stripes: wild *Oryza* and elite rice cultivars shape agricultural weed evolution in southeast Asia. Mol. Ecol. 23, 5003–5017. doi: 10.1111/mec.12922 25231087

[B229] Sudheesh S. Verma P. Forster J. W. Cogan N. O. I. Kaur S. (2016). Generation and characterisation of a reference transcriptome for lentil (*Lens culinaris* medik.). Int. J. Mol. Sci. 17. doi: 10.3390/ijms17111887 PMC5133886 27845747

[B1000] Sudheesh S. Rodda M. S. Davidson J. Javid M. Stephens A. Slater A. T. Cogan N. O.I.. (2016). SNP-Based Linkage Mapping for Validation of QTLs for Resistance to Ascochyta Blight in Lentil. Front. Plant Sci. 7, , 1604–16. doi: 10.3389/fpls.2016.01604 27853461 PMC5091049

[B230] Suvorova G. (2014). Hybridization of cultivated lentil *Lens culinaris* medik. and wild species *L. tomentosus* ladizinsky. Czech J. Genet. Plant Breed. 50, 130–134. doi: 10.17221/231/2013-CJGPB

[B231] Tahir M. Lindeboom N. Båga M. Vandenberg A. Chibbar R. (2011). Composition and correlation between major seed constituents in selected lentil (*Lens culinaris* medik) genotypes. Can. J. Plant Sci. 91, 825–835. doi: 10.4141/cjps2011-010

[B232] Tahir M. Muehlbauer, F. J. (1994). Gene mapping in lentil with recombinant inbred lines. J. Heredity 85, 306–310. doi: 10.1093/oxfordjournals.jhered.a111464

[B233] Tanksley S. D. McCouch S. R. (1997). Seed banks and molecular maps, unlocking genetic potential from the wild. Science 277, 1063–1066. doi: 10.1126/science.277.5329.1063 9262467

[B234] Tanyolac B. Ozatay S. Kahraman A. Muehlbauer F. J. (2010). Linkage mapping of lentil (*Lens culinaris* l.) genome using recombinant inbred lines revealed by AFLP, ISSR, RAPD and some morphologic markers. Sustain. Dev. 2, 001–006.

[B235] Tayşi N. Kaymaz Y. Ateş D. Sari H. Toker C. (2022). And tanyolac, M.B). complete chloroplast genome sequence of. Lens ervoides comparison to Lens culinaris. Sci. Rep. 12, 15068. doi: 10.1038/s41598-022-17877-7 36064865 PMC9445179

[B236] Temel H. Y. Göl D. Akkale H. B. K. Kahriman A. Tanyolaç M. B. (2015). Single nucleotide polymorphism discovery through illumina-based transcriptome sequencing and mapping in lentil. Turk. J. Agric. Forest. 39, 470–488.doi: 10.3906/tar-1409-70

[B237] Temel H. Y. Gol D. Kahriman A. Tanyolac M. B. (2014). “Construction of linkage map through genotyping-by-sequencing in lentil,” In Proceedings of plant and animal genome conference XXII, SanDiego, CA, P 358.

[B238] Thormann I. Endresen D. T. F. Rubio-Teso M. L. Iriondo M. J. Maxted N. Parra-Quijano M. (2014). Predictive characterization of crop wild relatives and landraces: Technical guidelines version 1. Biodiversity International, Rome. 1–40

[B239] Tito R. Vasconcelos H. L. Feeley K. J. (2018). Global climate change increases risk of crop yield losses and food insecurity in the tropical Andes. Glob Chang Biol. 24, e592–e602. doi: 10.1111/gcb.13959 29055170

[B240] Tiwari M. Singh B. Min D. Jagadish S. K. (2022). Omics path to increasing productivity in less-studied crops under changing climate-lentil a case study. Front. Plant Sci. 13. doi: 10.3389/fpls.2022.813985 PMC9125188 35615121

[B241] Toklu F. A. Karaköy T. Hakl E. Bicer T. Brandolini A. Kilian B.. (2009). Genetic variation among lentil (*Lens* culinaris medik) landraces from southeast Turkey. Plant Breed. 128, 178–186. doi: 10.1111/j.1439-0523.2008.01548.x

[B242] Tollefson J. (2020). How hot will earth get by 2100? Nature 580, 443–446. doi: 10.1038/d41586-020-01125-x 32322083

[B243] Tripathi K. Kumari J. Gore P. G. Mishra D. C. Singh A. K. Mishra G. P.. (2021). Agro-morphological characterization of lentil germplasm of Indian national genebank and development of a core set for efficient utilization in lentil improvement programs. Front. Plant Sci. 12. doi: 10.3389/fpls.2021.751429 PMC8828943 35154171

[B244] Tullu A. Banniza S. Bett K. Vandenberg A. (2011). A walk on the wild side: exploiting wild species for improving cultivated lentil. Grain Legume 56, 13–14.

[B247] Tullu A. Banniza S. Tar’an B. Warkentin T. Vandenberg A. (2010). Sources of resistance to ascochyta blight in wild species of lentil (*Lens* culinaris medik). Genet. Res. Crop Evol. 57, 1053–1063. doi: 10.1007/s10722-010-9547-7

[B245] Tullu A. Bett K. Banniza S. Vail S. Vandenberg A. (2013). Widening the genetic base of cultivated lentil through hybridization of‘Eston’ and Accession IG 72815. Can. J. Plant Pathol. 93, 1037–1047. doi: 10.4141/cjps2013-072

[B246] Tullu A. Buchwaldt L. Lulsdorf M. Banniza S. Barlow B. Slinkard A. E.. (2006a). Sources of resistance to anthracnose (*Colletotrichum truncatum*) in wild *Lens* species. Genet. Res. Crop Evol. 53, 111–119. doi: 10.1007/s10722-004-1586-5

[B248] Tullu A. Buchwaldt L. Warkentin T. Taran B. Vandenberg A. (2003). Genetics of resistance to anthracnose and identification of AFLP and RAPD markers linked to the resistance gene in PI 320937 germplasm of lentil (*Lens culinaris* medikus). Theor. Appl. Genet. 106, 428–434. doi: 10.1007/s00122-002-1042-x 12589542

[B249] Tullu A. Tar’an B. Breitkreutz C. Banniza S. Warkentin T. D. Vandenberg A.. (2006b). A quantitative-trait locus for resistance to ascochyta blight [*Ascochyta lentis*] maps close to a gene for resistance to anthracnose [*Colletotrichum truncatum*] in lentil. Can. J. Plant Pathol. 28, 588–595. doi: 10.1080/07060660609507337

[B250] Tullu A. Tar’an B. Warkentin T. Vandenberg A. (2008). Construction of an intraspecific linkage map and QTL analysis for earliness and plant height in lentil. Crop Sci. 48, 2254–2264. doi: 10.2135/cropsci2007.11.0628

[B251] Tyagi M. C. Sharma B. (1995). “Protein content in lentil (Lens culinaris medik.),” in Genetic research and education: Current trends and the next fifty years. Eds. Sharma B. Kulshreshtha V. P. Gupta N. Mishra S. K. (New Delhi: Indian Society of Genetics and Plant Breeding), 1031–1034.

[B252] Upadhyaya H. D. Reddy L. J. Gowda C. L. Singh S. (2006). Identification of diverse groundnut germplasm: Sources of early maturity in a core collection. Field Crops Res. 97, 261–271. doi: 10.1016/j.fcr.2005.10.010

[B253] Vail S. Strelioff J. V. Tullu A. Vandenberg A. (2012). Field evaluation of resistance to *Colletotrichum truncatum* in *Lens culinaris*, *Lens ervoides*, and *Lens ervoides* × *Lens culinaris* derivatives. Field Crops Res. 126, 145–151. doi: 10.1016/j.fcr.2011.10.002

[B254] Vaillancourt R. E. Slinkard A. E. (1993). Linkage de charactbres morphologiques et d’isoerzymes chez la lentifle. Can. J. Plant Sci. 73, 917–926. doi: 10.4141/cjps93-122

[B255] Van Oss H. Aron Y. Ladizinsky G. (1997). Chloroplast DNA variation and evolution in the *Lens* mill. Theor. Appl. Genet. 94, 452–457. doi: 10.1007/s001220050436

[B256] Verma P. Goyal R. Chahota R. K. Sharma T. R. Abdin M. Z. Bhatia S. (2015). Construction of a genetic linkage map and identification of QTLs for seed weight and seed size traits in lentil (*Lens culinaris* medik.). PloS One 10, e0139666. doi: 10.1371/journal.pone.0139666 26436554 PMC4593543

[B257] Verma P. Shah N. Bhatia S. (2013). Development of an expressed gene catalogue and molecular markers from the *de novo* assembly of short sequence reads of the lentil (*Lens culinaris* m edik.) transcriptome. Plant Biotechnol. J. 11, 894–905. doi: 10.1111/pbi.12082 23759076

[B258] Verma P. Sharma T. R. Srivastava P. S. Abdin M. Z. Bhatia S. (2014). Exploring genetic variability within lentil (*Lens* culinaris medik.) and across related legumes using a newly developed set of microsatellite markers. Mol. Biol. Rep. 41, 5607–5625. doi: 10.1007/s11033-014-3431-z 24893599

[B259] Vijayan P. Vandenberg A. Bett K. E. (2009)A mixed genotype lentil EST library representing the normalized transcriptome of different seed development stages. Available at: http://www.ncbi.nlm.nih.gov/nucest/?term=lens%20culinaris.

[B260] Vilayheuang K. Borrayo E. Kawase M. Watanabe K. N. (2020). “Development of Laos khao kai noi rice landrace (Oryza sativa l.) core collection as a model for rice genetic resources management in the Laos national genebank,” in IOP conference series: Earth and environmental science, vol. 482, 1. (IOP Publishing), 012039. doi: 10.1088/1755-1315/482/1/012039

[B261] Wang X. Chen L. Ma J. (2019). Genomic introgression through interspecific hybridization counteracts genetic bottleneck during soybean domestication. Genome Biol. 20, 1–15. doi: 10.1186/s13059-019-1631-5 30700312 PMC6354408

[B262] Wang D. Yang T. Liu R. Li N. Wang X. Sarker A.. (2020). RNA-Seq analysis and development of SSR and KASP markers in lentil (*Lens culinaris* medikus ssp. *culinaris*). Crop J. 8, 953–965. doi: 10.1016/j.cj.2020.04.007

[B263] Wong M. M. Gujaria-Verma N. Ramsay L. Yuan H. Y. Caron C. Diapari M.. (2015). Classification and characterization of species within the *Lens* using genotyping-by-sequencing (GBS). PloS One 10, e0122025. doi: 10.1371/journal.pone.0122025 25815480 PMC4376907

[B264] Yaqoob H. Tariq A. Bhat B. A. Bhat K. A. Nehvi I. B. Raza A.. (2023). Integrating genomics and genome editing for orphan crop improvement: a bridge between orphan crops and modern agriculture system. GM Crops Food 14 (1), pp.1–pp20. doi: 10.1080/21645698.2022.2146952 PMC9828793 36606637

[B265] Yonezawa K. Nomura T. Morishima M. (1995). “). sampling strategies for use in stratified germplasm collections,” in Core collections of plant genetic resources. Eds. Hodgkin T. Brown A. H. D. Hintum van T. Morales E. A. V. (Chichester: John Wiley and Sons), 35–53.

[B266] Yoshino K. Numajiri Y. Teramoto S. Kawachi N. Tanabata T. Tanaka T.. (2019). Towards a deeper integrated multi-omics approach in the root system to develop climate-resilient rice. Mol. Breed. 39, 165. doi: 10.1007/s11032-019-1058-4

[B267] Yuan H. Y. Caron C. T. Ramsay L. Fratini R. de la Vega M. P. Vandenberg A.. (2021). Genetic and gene expression analysis of flowering time regulation by light quality in lentil. Ann. Bot. 128, 481–496. doi: 10.1093/aob/mcab083 34185828 PMC8414921

[B268] Yuan H. Y. Saha S. Vandenberg A. Bett K. E. (2017). . Front. Plant Sci. 8. doi: 10.3389/fpls.2017.00386 PMC5359283 28377784

[B269] Zamir D. Ladizinsky G. (1984). Genetics of allozyme variants and linkage groups in lentil. Euphytica 33, 329–336. doi: 10.1007/BF00021129

[B270] Zeroual A. Baidani A. Idrissi O. (2023). Drought stress in lentil (*Lens culinaris*, medik) and approaches for its management. Horticulturae. 9 (1), 1–25. doi: 10.3390/horticulturae9010001

[B271] Zhang Y. Zhang X. Che Z. Wang L. Wei W. Li D. (2012). Genetic diversity assessment of sesame core collection in China by phenotype and molecular markers and extraction of a mini-core collection. BMC Genet. 13, 1–14. doi: 10.1186/1471-2156-13-102 23153260 PMC3574832

[B272] Zhao C. Liu B. Piao S. Wang X. Lobell D. B. Huang Y.. (2017). Temperature increase reduces global yields of major crops in four independent estimates. Proc. Natl. Acad. Sci. U.S.A 114, 9326–9331. doi: 10.1073/pnas.1701762114 28811375 PMC5584412

[B273] Zilli M. Scarabello M. Soterroni A. C. Valin H. Mosnier A. Leclère D.. (2020). The impact of climate change on brazil’s agriculture. Sci. Total Environ. 740, 139384. doi: 10.1016/j.scitotenv.2020.139384 32562983

[B274] Zohary D. Hopf M. (1988). Domestication of plants in the old world (London: Clarendon Press).

[B275] Zohary D. Hopf M. (2000). Domestication of plants in the old world: The origin and spread of cultivated plants in West Asia, Europe and the Nile valley (Oxford: Oxford university press).

